# A UPLC-Q-TOF-MS-Based Metabolomics Approach to Screen out Active Components in Prepared Rhubarb for Its Activity on Noxious Heat Blood Stasis Syndrome

**DOI:** 10.3389/fphar.2022.907831

**Published:** 2022-07-19

**Authors:** Hui Zhu, Yu Duan, Kunming Qin, Junjie Jin, Xiao Liu, Baochang Cai

**Affiliations:** ^1^ School of Pharmacy, Nanjing University of Chinese Medicine, Nanjing, China; ^2^ Engineering Center of State Ministry of Education for Standardization of Chinese Medicine Processing, Nanjing University of Chinese Medicine, Nanjing, China; ^3^ Nanjing Haichang Chinese Medicine Group Corporation, Nanjing, China; ^4^ Nanjing Haiyuan Prepared Slices of Chinese Crude Drugs Co., Ltd., Nanjing, China

**Keywords:** prepared rhubarb, metabolomics, UHPLC-Q-TOF-MS, noxious heat blood stasis syndrome, gray correlation analysis

## Abstract

**Background:** Prepared rhubarb was obtained by steaming raw rhubarb with wine. Different from raw rhubarb with a purgative effect, prepared rhubarb shows effects of promoting blood circulation and removing blood stasis. However, the mechanisms of its action through regulating endogenous metabolites remain unclear.

**Purpose:** The purpose of this study was to explore active chemical components in prepared rhubarb for its activity on noxious heat blood stasis syndrome (NHBS) by comprehensive metabolomics profiling.

**Study design:** Plant extracts usually show their activities in a synergistic way; therefore, integrated omics was developed as a rational way for a better understanding of their biological effects and potential active compounds.

**Methods:** The activities of prepared rhubarb were evaluated by biochemical and metabolomic analysis; meanwhile, serum chemical profiles were sought using UHPLC-Q-TOF-MS. Gray correlation analysis (GCA) was used for calculating the underlying correlations between them.

**Results:** The metabolomics profiles of rat plasma from model and control groups were significantly different, with 31 endogenous metabolites changed by NHBS. Then, after the administration of prepared rhubarb, 18 of them were regulated. Multiple metabolic pathways were disturbed after NHBS modeling and restored by prepared rhubarb, among which had a greater impact on sphingolipid metabolism. A total of 28 compounds from prepared rhubarb absorbed into the plasma were identified, including nine prototypes and 19 metabolites. Statistical results suggested that rhein and its metabolites accounted for half of the top 10 active compounds in prepared rhubarb for its biomedical activities.

**Conclusion:** This study presented evidence for the therapeutic effects and active chemicals of prepared rhubarb on NHBS in the way of metabolomics.

## Introduction

Rhubarb is a classical botanical drug derived from the root and rhizome of three species of the *Polygonaceae* family, namely, *Rheum palmatum* L., *Rheum tanguticum* Maxim. ex Balf., and *Rheum officinale* Bail., which has a large range of processed products to satisfy various clinical medications (Chinese Pharmacopoeia Commission Pharmacopoeia of the People’s Republic of China, 2020). Prepared rhubarb is obtained by steaming raw rhubarb with wine. This process can effectively relieve the original purgative effect of rhubarb and improve its efficacy in removing pathogenic heat and toxins from the organism, which could be applied for noxious heat blood stasis syndrome treatment. Noxious heat blood stasis syndrome (NHBS) is a common clinical complex syndrome caused by heat, and its pathogenesis is closely related to inflammation, fever, and blood stasis. Many inflammatory diseases and their complications such as viral hepatitis, cardiovascular disease, and systemic lupus erythematosus belong to the category of NHBS; the clinical treatment is mainly based on clearing heat and detoxifying, promoting blood circulation, and removing blood stasis (Zhang et al., 2019; Zhong et al., 2020; Xuan et al., 2022). Modern research showed that prepared rhubarb could cure diabetic bullae and infantile fester tonsillitis, which was in accordance with its characteristic of heat-clearing and detoxifying. In fact, whether it was applied singly or in combination with other kinds of botanical drugs for prescriptions, such as in Dahuang Zhechong Pill or Xiayuxue Decoction (Zhao et al., 2014; Dai et al., 2021; Li, 2021), prepared rhubarb exerted a good effect of promoting blood circulation. Because of the lowered purgative effect, the application of prepared rhubarb became much safer than raw rhubarb. So, prepared rhubarb was recorded in the list of “Functional Food Raw Materials” of China and could be given to children and old people for the treatment of diseases related to abnormal hemorheology in clinics (Wang et al., 2003; Wang et al., 2020; Zhao, 2020).

Spectrum-effect relationship analysis has become a classic method to explore the active compounds of traditional Chinese medicine (TCM) in recent years, by which the underlying correlation between chemical components and pharmacological functions of prepared rhubarb was explored in our previous study (Zhu et al., 2017). However, during the actual operation process, the acquisition of pharmacodynamics data was found to be quite difficult which showed strong uncontrollability. The detection of hemorheology and blood coagulation indicators could be easily affected by many factors including storage time, temperature, and so on. It was expected that all the samples could be detected as soon as possible within 2 h after their collection (Goyal et al., 2015; Komiyama, 2015; Denessen et al., 2020). However, the reality was that this kind of test was time-consuming work which usually led to a large number of samples being overstocked. Hence, it was difficult to ensure that all the samples collected could be tested within the time limitation. The operators must be well trained and experienced, otherwise, the accuracy of the results is hard to guarantee, and this would influence the objectivity and authenticity of the subsequent correlation analysis. Metabolomics is a subject to study the metabolic processes occurring in cells, tissues, and organisms, to reveal the metabolite information changes after the organism was disturbed. These small endogenous metabolites (biomarkers) produced in response to the environmental stimulus can be used to monitor the change in pathophysiological status. Nowadays, accumulating evidence shows that the occurrence of diseases and the intervention of drugs or functional foods would cause changes in the body’s metabolism (Xu et al., 2019; Bjerkhaug et al., 2021; Chen et al., 2021; Masoodi et al., 2021; Masutin et al., 2022). The advantage of omics analysis is that it provides a whole picture of the living organism, rather than focusing on a single known compound or compound group–“these analyses represent a more holistic approach as opposed to the investigation of a single protein or metabolite (Ulrich-Merzenich et al., 2007).” Compared to classical biochemical indexes, metabolomics biomarker information could be obtained using frozen plasma samples in storage; in addition, it could be detected by stable observation and quantization (Olivier, et al., 2019; Wörheide, et al., 2021). Therefore, in this study, biomarkers were introduced to replace the previous bio-effect indicators to improve the classic spectrum-effect correlation analysis, by which the controllability of data collection and the accuracy of subsequent analysis could be improved.

The liquid chromatography-mass spectrometry technique has been widely used in metabolomics at an exponentially increasing rate in recent years, owing to the following advantages: less consumption of analysis sample, high specificity, sensitivity, and accuracy of the instrument, and wide coverage of the metabolome (Zhao et al., 2018; Duan et al., 2020). As for the pharmacological activity of plant extracts, it may not be due to the effect of one or several major compounds; it usually may be a result of synergistic or antagonistic mechanisms. Even a low amount of phytochemical components may have an effect on this activity, but their identification and contribution are very difficult to ascertain. Here, an untargeted metabolomics study based on a UHPLC-Q-TOF-MS method was conducted to investigate the potential biomarkers of NHBS, and the modulating effects of prepared rhubarb on these metabolites. Based on the UHPLC-Q-TOF-MS technique, the identification of these phytochemicals at low abundance in plant extracts is no longer difficult. In addition, using integrated metabolomics coupled with the gray correlation analysis (GCA), the underlying relationship between the endogenous metabolites and the drug relative metabolites was calculated objectively. Finally, the active chemical components in prepared rhubarb for its activity on noxious heat blood stasis syndrome were screened out.

## Materials and Methods

### Materials and Reagents

Raw rhubarb was used as the material for the preparation of prepared rhubarb, which was purchased from Nanjing Haichang Chinese Medicine Group Corporation and authenticated by Prof. Jianwei Chen (School of Pharmacy, Nanjing University of Chinese Medicine). Raw rhubarb pieces of 100 kg were immersed for about 30 min in a mixed solution which comprised 30 kg glutinous rice wine and 30 kg water (10:3:3, *w*/*w*/*w*). After the liquid was absorbed exactly, the pieces were steamed for about 24 h until they turned black both inside and outside. Finally, they were naturally dried in a cool and dry condition. The decoction was prepared according to the classical literature, and the details were as follows: 180 g pieces of prepared rhubarb were immersed in water (1:10, *w*/*v*) for 30 min and boiled for 30 min, then the solution was filtrated through a four-layer mesh, after which the boiling process was repeated (1:8, *w*/*v*). Finally, the combined filtrate was concentrated to a density of 0.5 g/ml by rotary evaporation below 55°C for further experimental administration. The typical HPLC chromatogram of prepared rhubarb for chemical component profiling was provided as shown in Supplementary Figure S1 and the content of main components in the prepared rhubarb decoction is shown in the Supplementary Table S1.

Lipopolysaccharides (LPS) were purchased from Biosharp (Beijing, China). Adrenaline hydrochloride injections were acquired from Tianjin KingYork Group Co., Ltd. (Tianjin, China). HPLC-grade methanol and acetonitrile were obtained from E. Merck (Merck, Darmstadt, Germany). Formic acid of HPLC grade (99.9%) was purchased from Anaqua Chemical Supply (ACS, Houston, United States). Ultra-pure water was generated by using a Milli-Q water purification system (Millipore Corporation, Bedford, MA, United States). The TT (thrombin time), PT (prothrombin time), APTT (activated partial thromboplastin time), and FIB (fibrinogen) test kits were purchased from Steellex Science Instrument Company (Beijing, China). All the other chemicals were of analytical grade and purchased from Nanjing Chemical Reagent Company (Nanjing, China).

### Animals and Drug Administration

Male pathogen-free Sprague–Dawley rats, weighing 250 ± 20 g, were provided by the Slaccas Experiment Animal Company (Shanghai, China. Certificate No.: SCXK-2014-0001). All the rats were allowed to acclimate to the experimental environment (temperature of 22 ± 2°C; relative humidity of 40–60%) for at least 7 days before the experiment. Free access to food and water was allowed at all times except for fasting for 12 h before the experiment. All animal experiments conformed to the Guidelines for Animal Ethics Committee of the Nanjing University of Chinese Medicine.

The rats were randomly divided into three groups: control group, model group, and treatment group. The treatment group was further divided into six subgroups according to the point-in-time of blood collection (*n* = 6). The construction of the NHBS model of noxious heat blood stasis syndrome took 2 days: the rats were intraperitoneally injected with LPS (2 mg/kg) on day 1 and injected with 0.1% adrenaline hydrochloride (0.6 mg/kg) twice in a hypodermic way with a 4 h interval on day 2. The rats in the control group were injected with normal saline in the same manner. Also, the related evaluation indexes were detected to confirm the results of modeling. After successful modeling, rats in the treatment group were orally administrated with prepared rhubarb decoction (5 g/kg), whereas rats in the other groups were given the same volume of water instead. The abdominal aortic blood samples were collected at 0.5, 1, 2, 4, and 8 h after treatment on day 3 and 0.5 h after administration on day 4, and were recorded as group T1-T6. Blood samples were centrifuged at 3000 rpm/min for 10 min, and then the supernatant liquor of each sample was divided into two parts: one part was used for biochemical analysis; the other was transferred into 1.5 ml polypropylene tubes and stored under −80°C for further UPLC-Q-TOF-MS analysis.

### Instruments and UHPLC-Q-TOF-MS Conditions

The analysis work was performed on a UHPLC-Q-TOF-MS system (Shimadzu, Kyoto, Japan), which has the following units: an LC-30A binary pump, an autosampler (Model SIL-20ACXR), an online degasser (DGU-20A5R), a column temperature controller compartment (CTO-30A), and a hybrid quadrupole time-of-flight tandem mass spectrometer (AB Sciex, Concord, ON, United States). An automatic coagulation analyzer (Beijing Zonic Technology Development Co., Ltd, China) was used to detect the plasma viscosity and the whole blood viscosity at four shear rates. An LG-PABER-1CH coagulation analyzer (Beijing Steellex Science Instrument Company, China) was used for the detection of TT, PT, APTT, and FIB.

The separation was achieved on an Extend C_18_ column (Agilent, United States, 2.1 mm × 100 mm, 1.8 µm). The column temperature was maintained at 30°C. The mobile phase comprised 0.1% aqueous formic acid (A) and acetonitrile (B) at a constant flow rate of 0.3 ml/min. The gradient elution program was as follows: 0–3 min, 5–20% B; 3–7 min, 20–80% B; 7–30 min, 80–90% B; 30–32 min, 90–5% B. Then the column was reconditioned at 5% B for 3 min to prepare for the next injection. The samples were stored in an autosampler at 4°C before injection and the injection volume was 2 µl.

The MS instrument was equipped with an ESI ion source operating in both positive and negative ion modes. The optimized source temperature (TEM) and ion spray voltage (IS) were set at 550°C and −5500 V, respectively. The declustering potential was set at 60/−60 with a collision energy of 30/−30 V. The curtain gas (CUR), ion source gas 1 (GAS 1), and ion source gas 2 (GAS 2) were 35 psi, 55 psi, and 55 psi, respectively. The mass range was scanned from 100 to 2000 Da in the TOF MS mode and 50 to 1000 in the TOF MS/MS mode, respectively. The experiments were run with 200 *ms* accumulation time for TOF MS and 80 *ms* accumulation time for TOF MS/MS. Automatic calibration was carried out at intervals of every six samples during the analytical run, to evaluate the stability and analytical repeatability of the instrument. Information-dependent acquisition (IDA) with dynamic background subtraction (DBS) was used to complete the data acquisition of low signal levels by reducing the influence of matrix interference.

### Sample Pretreatment for UHPLC-Q-TOF-MS Analysis

All frozen plasma samples were thawed at 4°C. A total of 100 µl of the plasma sample was added with 300 µl of acetonitrile, then each mixture was vortexed for 3 min and centrifuged at 13,000 rpm/min for 5 min. The supernatant was transferred into an autosampler vial and stored at 4°C.

### Data Acquisition and Processing

A total of 400 µl of blood or plasma was injected for the determination of whole blood viscosity or plasma viscosity, respectively. The levels of TT, PT, APTT, and FIB in rat serums were measured following the manufacturer’s instruction, and all the results were expressed as mean ± SD. It is worth noting that the plasma and reagent need to be mixed and preheated for 3 min before APTT and FIB detection.

After using analyst software to control the equipment and collect raw data, the chromatographic peaks were processed by MarkerView software with the following parameters: retention time range within 1–30 min, mass scan range within 100–1000 Da, tolerance range of 0.01 Da and, peak intensity threshold of 100. The extracted ionic intensity was normalized by the total peak area method. Then, the normalized data were imported into SIMCA-P software for the PCA analysis (unsupervised pattern recognition) to have a preliminary understanding of the most realistic metabolic differences between the groups. OPLS-DA analysis (supervised mode) was further performed to distinguish the overall differences in metabolic profiles between groups, and cumulative *R* ^*2*^ *Y* and *Q* ^*2*^ values were calculated to evaluate the fitness and predictive capability of constructed pattern recognition models. The variables differentially expressed between the groups were screened out and selected as candidate endogenous metabolites based on the variable importance in projection (VIP) of the OPLS-DA analysis and *p*-value (VIP>1, *p* < 0.05). Afterward, corresponding structures were obtained by searching online public databases such as HMDB and KEGG. The potential endogenous metabolites were finally identified by checking the accurate molecular weight and secondary MS/MS spectrum. The contents of endogenous metabolites in plasma were expressed as the intensity to exhibit the variation trends among different groups. The generation of a heatmap and the analysis of the involved metabolic pathways were completed by the dedicated website MetaboAnalyst.

In our previous study, a database of chemical compounds in botanical drugs belonging to rheum and all varieties of processed rhubarb has already been established, so compounds and structural characteristics in the prepared rhubarb decoction were thus identified. Based on this, the information of compounds and their possible metabolic pathways (including I phase and II phase metabolism) were imported into MetabolitePilot software, by which both prototypes and metabolic compounds were predicted by the functions of generic peak finding, predicted metabolites, isotope pattern, and mass defect. PeakView software could link the chemical structure to MS/MS spectrums (matching rate >75% and the *m/z* error within 10 ppm) to complete the final identification of these compounds based on their accurate molecular weights and the matching rates of the reasonable fragmentations. Ions in different samples that showed the same retention time (tolerance of 0.2 min) and the same *m/z* value could be defined as the same ions. The spectrum of the rat plasma in the model group was selected as the control to eliminate chromatographic peak interference coming from the endogenous compounds.

To guarantee the accuracy and reproducibility of the results, the compounds in plasma samples with an intensity below 100 were not taken into account. The intensity of compounds absorbed into rat plasma at different time points was the average value measured by data from six individuals.

### Gray Correlation Analysis

The gray correlation analysis method can significantly reduce the bias caused by information asymmetry, hence, it was widely used to investigate the correlation degree between two sets of variations with a relatively simple calculation way (Hou et al., 2020; Ren, et al., 2020). The gray correlation degree was calculated by Excel functions and its data analysis tool. In detail, the dimensionless processed sequence is set as the feature sequence *X*_*0*_= (*x*_*0*_ (1), *x*_*0*_ (2), ..., *x*_*0*_ (*n*)), then the sequence of associated factors is determined as follows: *X*_*1*_= (*x*_*1*_ (1), *x*_*1*_ (2), ..., *x*_*1*_ (*n*)), *X*_*2*_ = (*x*_*2*_ (1), *x*_*2*_ (2), ..., *x*_*2*_ (*n*)), …... *X*_*i*_ = (*x*_*i*_ (1), *x*_*i*_ (2), ..., *x*_*i*_ (*n*)), and the correlation coefficient could be calculated by the following formula:

$$\xi \left(x_{0}\left(k\right),\;x_{i}\left(k\right)\right)=\;\frac{\underset{i}{\min}\underset{k}{\min}\;\left|x_{0}\left(k\right)-x_{i}\left(k\right)\right|\;+\;\rho \;\underset{i}{\max}\;\underset{k}{\max}\;\left|x_{0}\left(k\right)-x_{i}\left(k\right)\right|}{\left|x_{0}\left(k\right)-x_{i}\left(k\right)\right|\;+\;\rho \;\underset{i}{\max}\;\underset{k}{\max}\;\left|x_{0}\left(k\right)-x_{i}\left(k\right)\right|}$$

*ρ* is the distinctive coefficient lying between 0 <*ρ* < 1, and it is usually set as 0.5.

The gray correlation grade could finally be obtained by averaging the correlation coefficient at different time points:

$$\xi \left(X_{0},\;X_{i}\right)=\;\frac{1}{n}\sum_{k=1}^{n}\xi \left(x_{0}\left(k\right)-x_{i}\left(k\right)\right)$$

### Construction of a Compound-Target-Pathway-Disease Network

Based on the compounds screened out, related targets were found by searching the TCMSP database ((http://tcmspw.com/tcmsp.php), consulting the literature, eduction by the Swiss Target Prediction database (http://www.swisstargetprediction.ch/)), and further inputting to UniProt (https://www.uniprot.org/) identifiers. The main pathways and associated diseases were extracted from the Integrated Discovery database (https://david.ncifcrf.gov/). The compound-target-pathway-disease network was finally constructed by using Cytoscape software.

## Results

### Evaluation of Therapeutic Effects of Prepared Rhubarb

**FIGURE 1:**
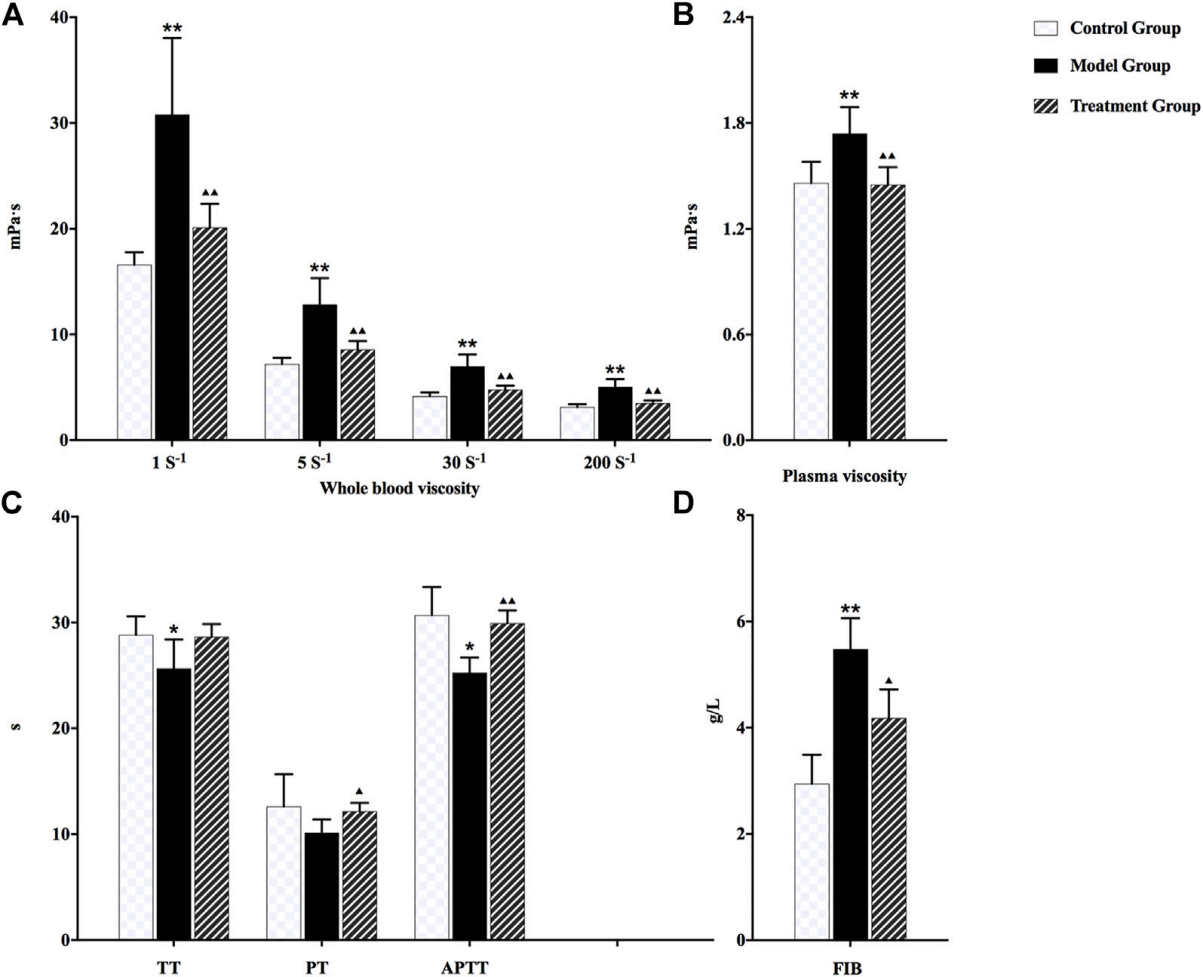
Changes in each pharmacodynamic index of rats in different groups: **(A)** whole blood viscosity, **(B)** plasma viscosity, **(C)** TT, PT, and APTT, and **(D)** FIB. ^▲▲^ *p* < 0.01 and ^▲^ *p* < 0.05 compared with the NHBS model group; ^*^ *p* < 0.05 and ^**^ *p* < 0.01 compared with the control group.

As shown in Figure 1, the whole blood viscosity, plasma viscosity, and content of plasma fibrinogen in the model group were significantly increased compared with rats in the control group. However, TT, PT, and APTT values were decreased. The obvious microcirculation disturbance and hemorheology abnormality were observed in rats after modeling, indicating that noxious heat blood stasis syndrome was constructed successfully. After the administration of prepared rhubarb, all the aforementioned pharmacodynamic indexes were reverse-regulated in varying degrees, suggesting an exact therapeutic effect on NHBS model rats.

### Metabolic Spectrum Analysis

**FIGURE 2:**
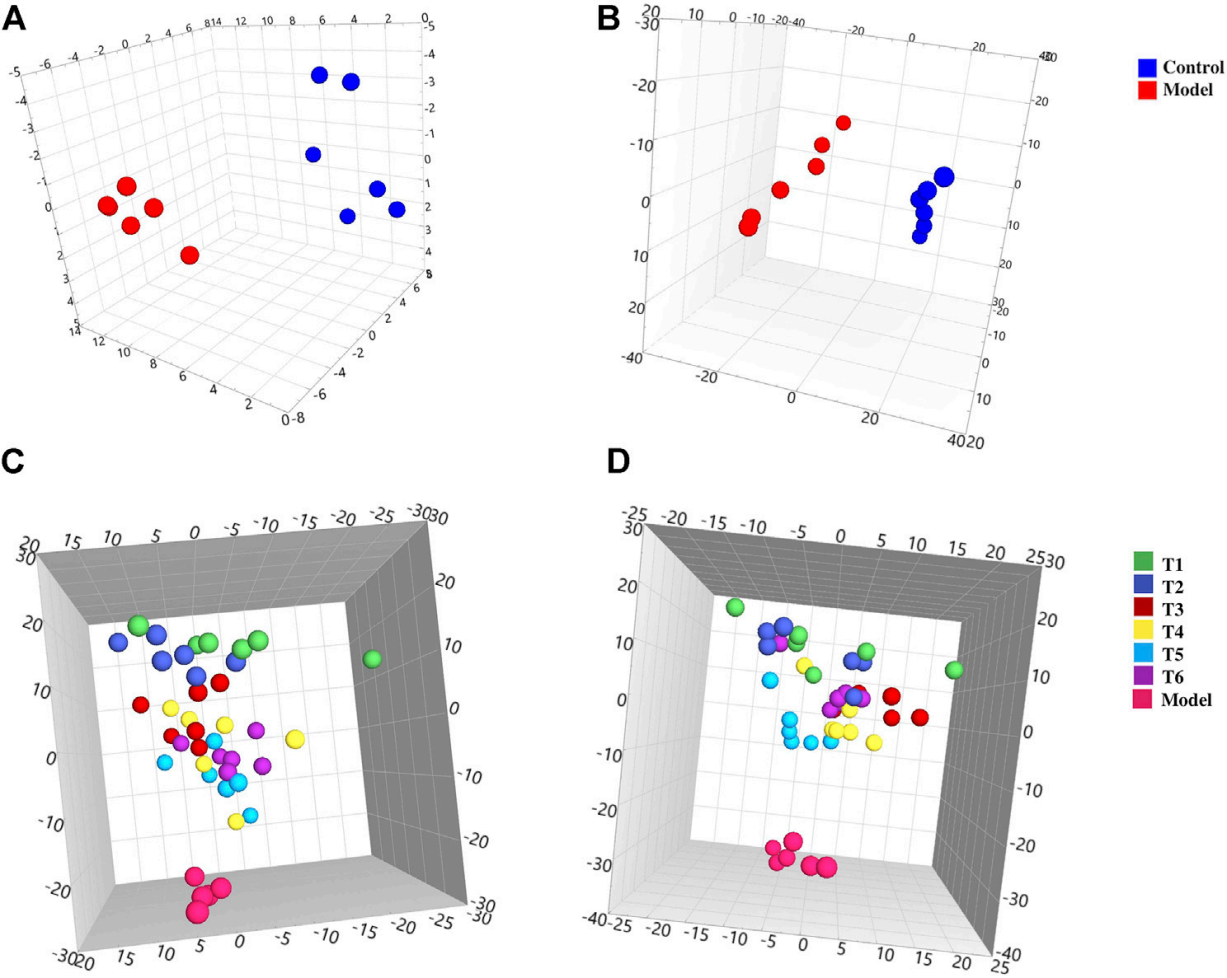
3D PCA score plots of the plasma samples from different groups. Control and model groups: **(A)** ESI^+^, *R* ^*2*^ *X* = 0.796, *Q* ^*2*^ = 0.754 and **(B)** ESI^−^, *R* ^*2*^ *X* = 0.920, *Q* ^*2*^ = 0.838; model and treatment groups: **(C)** ESI^+^, *R* ^*2*^ *X* = 0.730, *Q* ^*2*^ = 0.559 and **(D)** ESI^−^, *R* ^*2*^ *X* = 0.752, *Q* ^*2*^ = 0.552.

The plasma samples were analyzed by the UHPLC-Q-TOF-MS method in both positive and negative iron modes. The representative total ion chromatograms are shown in Supplementary Figure S2, and the extracted data were used for multivariate statistical analysis. PCA analysis was first taken into consideration to reflect the real differences between groups (Supplementary Figure S3). As shown in Figures 2A,B, a remarkable aggregation within the groups and an evident separation between model and control groups were observed, which indicated that the NHBS model was successfully constructed; meanwhile, the normal physiological metabolism of rats was affected after injection of LPS and adrenaline hydrochloride. To facilitate screening of candidate endogenous metabolites, OPLS-DA was further used to maximize the differences between the groups (Supplementary Figure S4). The candidate endogenous metabolites were excavated based on the criteria of a VIP value greater than 1 and *p-*value less than 0.05, and further identified according to practical and theoretical fragment ions provided by available databases. Consequently, a total of 31 endogenous metabolites in plasma were screened out by a comparison of the control group with the model group, including eight metabolites increased and 23 metabolites decreased, which could be used as potential biomarkers for noxious heat blood stasis syndrome indication. As listed in Supplementary Table S2, most of the metabolites detected belonged to lysophosphatidylcholine (*n* = 11), lysophosphatidyl ethanolamine (*n* = 4), sphingolipids (*n* = 3), unsaturated fatty acids (*n* = 3,) and amino acids (*n* = 2), suggesting that the occurrence of noxious heat blood stasis syndrome was mainly connected with multiple metabolic pathways including glycerophospholipid metabolism, sphingolipid metabolism, biosynthesis of unsaturated fatty acids, arachidonic acid metabolism, and aminoacyl-*t*RNA biosynthesis.

Similarly, the data of rat plasma from the control and model groups were subjected to both PCA (Supplementary Figure S5) and OPLS-DA (Supplementary Figure S6) analysis. Figures 2C,D show that scattered points representing rats in the model and different treatment groups were well separated, implying that prepared rhubarb had a certain intervention effect on the rat’s metabolism. It could be seen that sports of the T1 group were the farthest from those of the model group. Then samples at subsequent time points were found gradually approaching the model group with obvious overlaps between subgroups, which, in accordance with our previous findings, was that prepared rhubarb works quickly after administration while its curative effect would gradually become weak over time. Also, the samples in the T6 group showed an obvious callback trend, suggesting that continuous administration of prepared rhubarb was necessary to consolidate its curative effect. Based on the established OPLS-DA model with good applicability and high predictability (Supplementary Figure S7), a total of 21 endogenous metabolites were screened out, comprised of 5 decreased and 16 increased. The details and pathways involved are shown in Supplementary Table S3.

**FIGURE 3:**
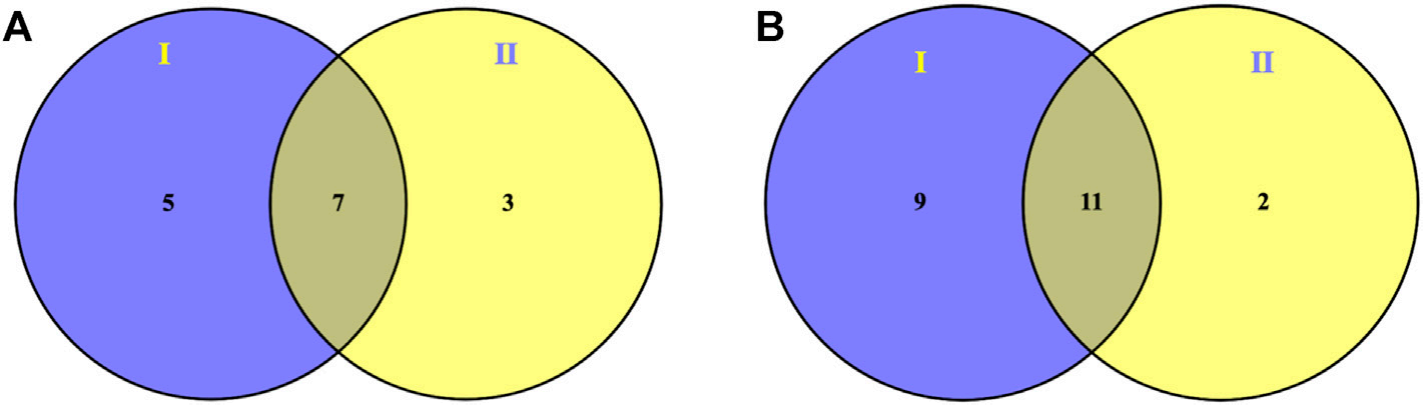
Venn diagrams showing overlapped modulation of prepared rhubarb on endogenous metabolites of NHBS rats in both **(A)** ESI^+^ and **(B)** ESI^−^ mode by pairwise analysis: I (potential endogenous metabolites of NHBS) versus Ⅱ (modulation of prepared rhubarb on endogenous metabolites of NHBS rats).

To further reveal the intervention effect of prepared rhubarb on NHBS rats from the perspective of the metabolic profile, pairwise analysis was performed by Venn diagrams. As shown in Figure 3, 18 endogenous metabolites were finally retained under positive and negative ion detective modes, which expressed significant differences after modeling but were reversely regulated after drug administration. They were L-isoleucine (leucine), L-phenylalanine, L-tryptophan, isovalerylcarnitine, sphinganine, phytosphingosine, eicosapentaenoic acid, sphingosine-1-phosphate (S1P), 11-octadecenylcarnitine, lysoPC (15:0), LysoPC (16:0), LysoPC (18:1), LysoPC (18:2), LysoPE (0:0/20:2), lysoPC (20:4), lysoPC (20:5), lysoPE (0:0/20:1), and lysoPE (0:0/22:4).

**FIGURE 4:**
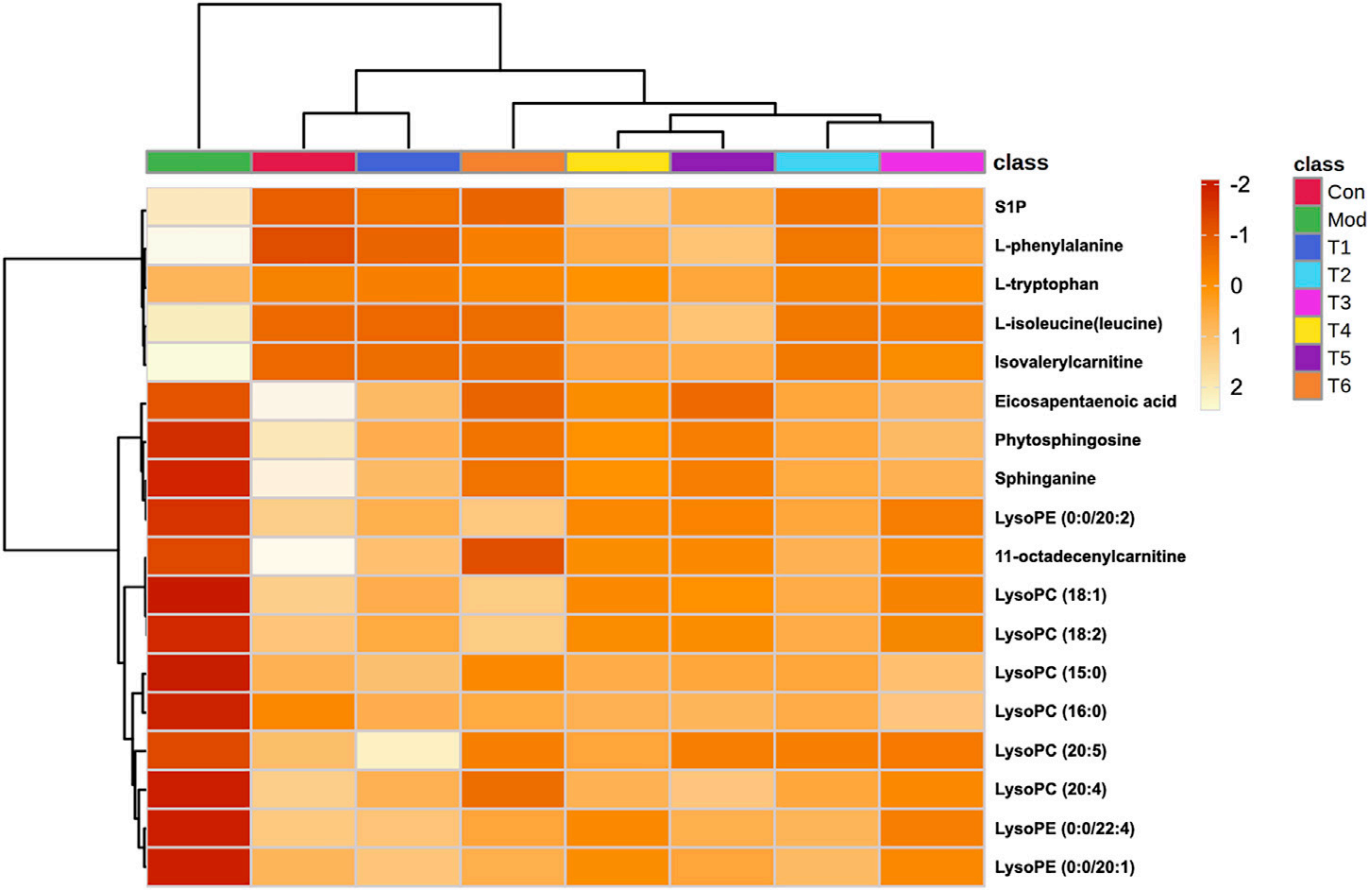
Heatmap with hierarchical clustering analysis showed the change in the biomarkers between each group.

As a supplement to verification, heatmaps were presented according to the extracted peak intensity data to observe the variation trends of these metabolites in the samples from different groups directly (Figure 4). As shown, the clustering analysis grouped all of these samples into two major distinct clusters, which exactly conformed to the different physiological and pathological statuses of rats. In addition, the T1 and control groups were classified into the same cluster, which was consistent with the result of the PCA. After dosing, 13 declined endogenous metabolites exerted varying degrees of increase; meanwhile, the level of five elevated endogenous metabolites was decreased. Given all of the aforementioned data, these 18 variables could be chosen as the characteristic indexes for the subsequent GCA analysis.

Correspondingly, prepared rhubarb could regulate the following pathways disturbed by NHBS: aminoacyl-*t*RNA biosynthesis; sphingolipid metabolism; valine, leucine, and isoleucine biosynthesis; valine, leucine, and isoleucine degradation; phenylalanine, tyrosine, and tryptophan biosynthesis; phenylalanine metabolism; biosynthesis of unsaturated fatty acids; glycerophospholipid metabolism; and tryptophan metabolism. These might lay a foundation for further exploration of drug action mechanisms.

### Analysis and Identification of Prepared Rhubarb Relative Compounds in Rat Plasma

Finally, 28 compounds from prepared rhubarb in rat plasma were identified, including nine prototype compounds and 19 metabolites. The detailed information, including their specific molecular formula, molecular weight, retention time, mass deviation, fragmentation, and matching rate are shown in Supplementary Table S4. Specific metabolic pathways of the 19 metabolites were concluded as glucuronidation, sulfation, hydroxylation, methylation, oxidation, hydroxylation + sulfation, decarboxylation, decarbonylation, hydroxylation + glucuronidation, and hydrolyzation. The proposed metabolic pathways of rhein in NHBS rat plasma were illustrated as an example (Supplementary Figure S8).

**FIGURE 5:**
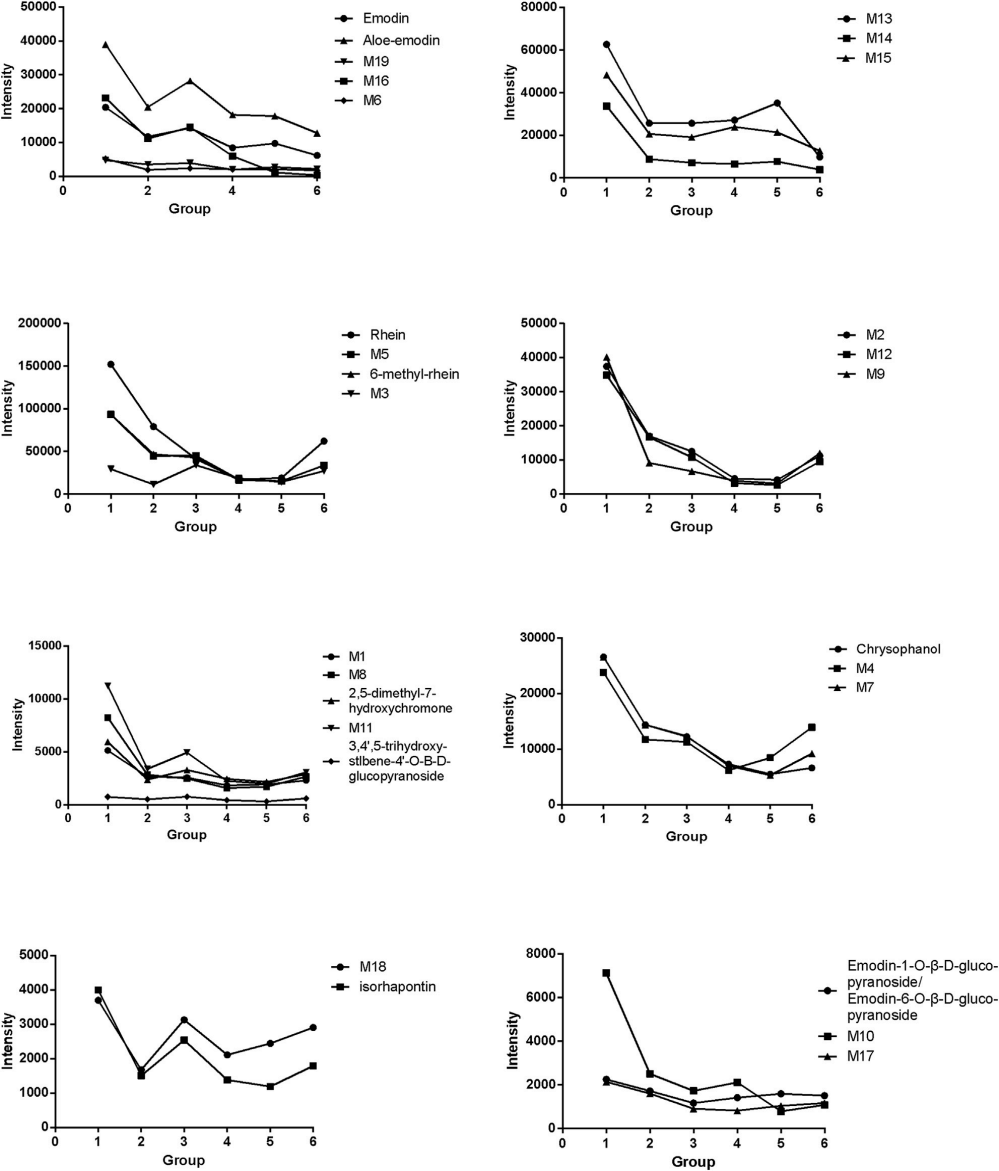
Intensity changes in compounds absorbed into the plasma of rats with noxious heat blood stasis syndrome after administration of prepared rhubarb decoction.

The variation trend of each compound is presented as intensity-time curves in Figure 5. It could be found that although the absorption process of each compound *in vivo* was different, similar trends could still be found among them.

### Gray Correlation Analysis Results

The results of the correlation between the intensity of each compound absorbed into rat plasma and the characteristic endogenous metabolites screened out are shown in Supplementary Table S5 (details listed in Supplementary Table S6). According to the score ranking, the top 10 potential active compounds in prepared rhubarb for its activity on noxious heat blood stasis syndrome were found to be chrysophanol monosulfate (M14), hydroxy-emodin glucuronide (M11), rhein glucuronide (M3), oxidative-rhein (M6), 1, 8-dihydroxy-anthraquinone (M10), aloe-emodin glucuronide (M13), hydroxy-emodin monosulfate (M8), hydroxy-rhein monosulfate (M1), emodin-1-*O-β*-D-glucopyranoside-desaturation (M15), and rhein. It is worth noting that rhein was the only prototype compound from prepared rhubarb among these 10 compounds, and the other nine were conjugate metabolites from chrysophanol, emodin, aloe-emodin, and rhein, which indicated that rhubarb anthraquinones were the main active compounds to exert the effects of prepared rhubarb in promoting blood circulation and removing blood stasis. Compared with our previous study, rhein glucuronide and hydroxy-emodin glucuronide still scored higher, and the contribution of rhein and its metabolites during the treatment cannot be ignored (Zhu et al., 2017).

**FIGURE 6:**
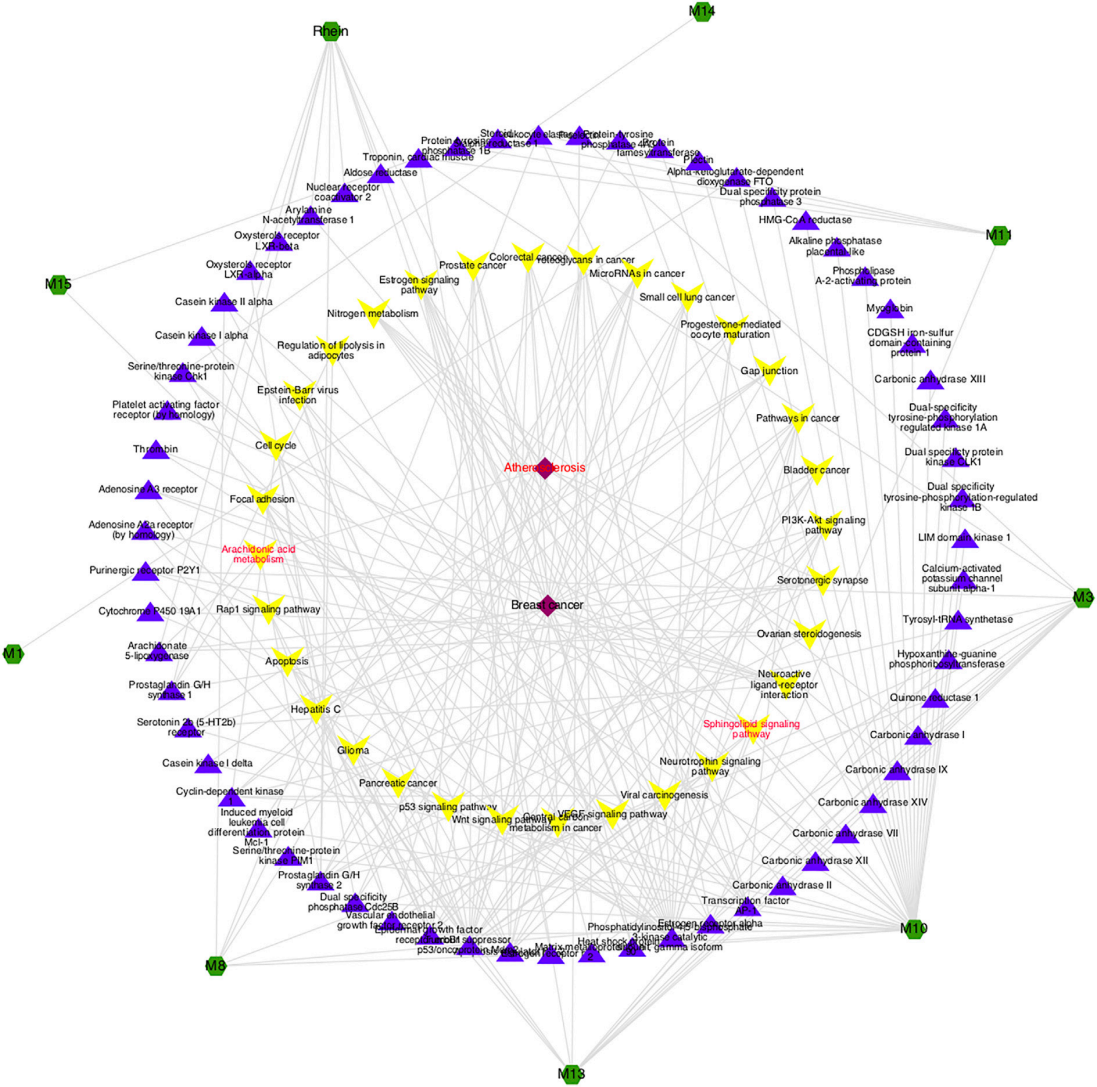
Potential compound-target-pathway-disease network. The green nodes represent potential active components, purple nodes represent related targets, yellow nodes represent pathways, and red nodes represent predicted diseases.

According to GCA results, the top 10 potential active compounds were selected for bioinformatics analysis as supplementary verification, also the compound-target-pathway-disease network was constructed. As shown in Figure 6, atherosclerosis is a common kind of cardiovascular and cerebrovascular disease in clinics, which belongs to the category of “blood stasis” in traditional Chinese medicine, and these compounds might exert a therapeutic effect by regulating various metabolic pathways including the sphingolipid signaling pathway and the arachidonic acid metabolism pathway. Part of the predictions were not only consistent with the results of the metabolomics but also in line with the efficacy and clinical application of prepared rhubarb (Liu et al., 2020; Guo et al., 2021), suggesting that the strategy based on metabolomics to find the active chemicals of TCM described in this study was quite feasible, giving reliable conclusions .

**TABLE 1:** Molecular docking scores.

Gene	P1	M1	M3	M8	M10	M11	M13	M14	M15
TP53	−76.02	−85.18	−105.66	−86.23	0	−100.14	−92.89	−74.50	−85.34
CASP3	−108.10	−112.634	−134.44	−110.67	−92.22	−128.38	−143.27	−108.22	−135.93
MAPK8	−110.39	−120.17	−134.53	−118.68	−97.97	−136.89	−138.00	−120.00	−136.91
MMP9	−87.10	−92.11	−129.32	−97.97	−76.80	−127.50	−131.15	−91.02	−101.63
EGFR	−95.17	−111.64	−138.76	−107.21	−85.17	−142.38	−136.43	−100.67	−128.05
ESR1	−96.48	0	−84.18	−105.97	−82.29	0	−107.17	−100.207	−110.59

The data represent the binding energy of the interaction (unit: kcal·mol-1); the larger the absolute value, the stronger the affinity; 0 means no interaction.

The pharmacological activity observed in functional plants was usually not due to a single compound. In most cases, plant extracts showed their activities based on synergistic or antagonistic effects. Therefore, the idea of a holistic approach described here was rational. Also, it was a remarkable fact that either the analysis of prototype components or these metabolites alone could not obtain the aforementioned ideal results, which prompted a synergistic work conducted by both prototype compounds and their metabolites. The verification of the efficacy of single or multiple potential active compounds and their precise mechanism of action still need to be further explored, especially the difficulty of obtaining metabolites and tracking their intracorporal process *in vivo*.

## Discussion

In this study, metabolomics was used to clarify that the metabolism profiles in noxious heat blood stasis syndrome rats changed significantly, and the disordered endogenous metabolites were excavated. Interestingly, most of the differential metabolites belonged to lipid molecules, the phenomenon of significant changes in lipid composition and expression in rats after modeling implied that the occurrence and development of noxious heat blood stasis syndrome were closely related to abnormal lipid metabolism, and the lipidomics method can be further applied to focus on the changes in lipid molecules of rats before and after the intervention of prepared rhubarb accurately.

Phytosphingosine, sphinganine, and sphingosine-1-phosphate are metabolites of sphingolipids, which are essential components of cell membranes and take part in many critical processes of signal transduction (Petersen et al., 2013; Tidhar et al., 2014; Drexler et al., 2021). Among them, phytosphingosine is the basic unit for the synthesis of various complex sphingolipid derivatives, which can protect the epidermal lipid barrier from external damage; sphinganine is involved in regulating the fluidity of lipid bilayer membranes, and both of them are closely related to changes in physiological and pathological states such as vascular growth, maturation, injury, and repair. After modeling, the level of phytosphingosine and sphinganine in noxious heat blood stasis syndrome rats significantly decreased, which may be due to their increased consumption under the blood stasis state. In addition, studies have shown that emodin in rhubarb can significantly reduce the expression of phytosphingosine and sphinganine in the serum of chronic constriction injury model rats to inhibit neuroinflammation, suggesting that emodin may be the main component involved in the regulation of sphingosine metabolism (Chen et al., 2022). Sphingosine 1-phosphate is generated by the phosphorylation of sphingosine catalyzed by SPHK1/SPHK2 and expressed on endothelial cells, which are involved in mediating vascular maturation and maintaining vascular integrity. S1P is released by activated platelets and exposes fibrinogen receptors by binding to platelet surface receptors, increasing platelet reactivity and further stimulating platelet aggregation, thus resulting in blood stasis (Jung et al., 2012; Parham et al., 2015). However, highly expressed S1P and FIB in noxious heat blood stasis syndrome rats were significantly downregulated after the intervention treatment, implying that prepared rhubarb can regulate sphingolipid metabolism to improve blood stasis.

LysoPC and LysoPE are both lysophospholipids. The 15 differential lysophospholipids screened in this experiment can also be regarded as potential biomarkers for noxious heat blood stasis syndrome. Consistent with the results of our experiment, recent studies have shown that lysophospholipids are related to the occurrence of inflammatory reactions and the maintenance of vascular endothelial function, which can be used to predict the occurrence of cardiovascular diseases such as atherosclerosis and stroke with high accuracy (Liu et al., 2017; Paapstel et al., 2018; Wang et al., 2021).

Arachidonic acid is stored in cell membrane phospholipids and is enzymatically released from these phospholipids by the action of the PLA2 enzyme. PGH_2_ catalyzed by cyclooxygenase is further catalyzed by specific prostaglandin synthase to obtain different forms of prostaglandins, which play an important role in the regulation of inflammation and oxidative stress. Among them, the synthesis and release of PGE_2_ catalyzed by the PGES enzyme can cause an increase in body temperature. PGI_2_ and TXA_2_, which are catalyzed by the PGIS and TXS enzymes, have opposing roles in regulating blood vessel and platelet states, respectively. They are rapidly converted into metabolites TXB_2_ and 6-keto-PGF_1α_ due to their extremely short half-lives. The broken dynamic balance between them leads to vasoconstriction and increased blood viscosity (Liu et al., 2019; Imig., 2020; Li et al., 2021). Similarly, the experimental results showed that prepared rhubarb can significantly reverse the level of arachidonic acid in noxious heat blood stasis syndrome rats. Meanwhile, the previous research by our group proved that prepared rhubarb can effectively correct the ratio of TXB_2_ and 6-keto-PGF_1α_ to normal in noxious heat blood stasis syndrome rats. It is indicated that prepared rhubarb plays the role of promoting blood circulation and removing blood stasis by regulating inflammatory response and improving blood rheology. In addition, studies have confirmed that a variety of prototype and derivative compounds in rhubarb are involved in the regulation of the synthesis and release of prostaglandins to exert corresponding pharmacological effects. For example, rhein can inhibit inflammatory factors such as IL-6, TNF-α, and PEG_2_ to reduce fructose-induced inflammatory cell erosion (Dong, 2016); emodin can reduce the release of arachidonic acid metabolites such as TXB and PGs by inhibiting PLA2 and 5-LOX, thus exerting anti-inflammatory effects (Peng et al., 2017); and chrysophanol-8-*O*-glucoside can significantly inhibit platelet aggregation and TXA2 of rats *in vitro* (Seo et al., 2012); however, the regulatory effects of other potential active ingredients screened in this study on the arachidonic acid metabolic pathway remain to be further explored.

## Conclusion

This was the first study in which the metabolomics profiles of NHBS were correlated with prototypes and metabolites from prepared rhubarb absorbed into rats’ plasma. In this study, a UHPLC-Q-TOF-MS method coupled with the GCA analysis was developed, by which the contribution of all molecules to the functions of prepared rhubarb in promoting blood circulation and removing blood stasis was calculated. The biomarker-based method described was proved to be a simple and reproducible characterization approach, which could provide a reference leading to the discovery of new compounds with a specific activity in TCM.

## Data Availability

The datasets presented in this study can be found in online repositories. The names of the repository/repositories and accession number(s) can be found in the article/Supplementary Material.

## Abbreviations

APTT, activated partial thromboplastin time; CUR, curtain gas; DAVID, database for annotation, visualization, and integrated discovery; DBS, dynamic background subtraction; FIB, fibrinogen; GAS1, ion source gas 1; GAS2, ion source gas 2; GCA, gray correlation analysis; IDA, information dependent acquisition; IS, ion spray voltage; LPS, lipopolysaccharides; NHBS, noxious heat blood stasis syndrome; PCA, principal component analysis; PT, prothrombin time; TCM, traditional Chinese medicine; TEM, source temperature; TT, thrombin time; UPLC-Q-TOF-MS, ultra-performance liquid chromatography coupled with time of flight mass spectrometry.
