# Supplementary material for: A UPLC-Q-TOF-MS-Based Metabolomics Approach to Screen out Active Components in Prepared Rhubarb for Its Activity on Noxious Heat Blood Stasis Syndrome
Source: Front Pharmacol. 2022 Jul 19;13:907831. doi: 10.3389/fphar.2022.907831 (PMC9343851; doi:10.3389/fphar.2022.907831)
Supplement: Supplementary file 1 [file Table1.DOCX]

## Supplementary Material

# Supplementary Tables

# Supplementary Figures

**Supplementary Table 1.** The content of main components in prepared rhubarb decoction.

| Component name | Concentration (mg/g) |
| --- | --- |
| Gallic acid | 2.641 ± 0.015 |
| Aloe emodin | 0.094 ± 0.001 |
| Rhein | 0.387 ± 0.003 |
| Emodin | 0.120 ± 0.002 |
| Chrysophanol | 0.109 ± 0.002 |
| Physcion | 0.034 ± 0.001 |

**Supplementary Table 2.**Potential endogenous metabolites in plasma from noxious heat blood stasis syndrome rats and their identification results.

| Ion mode | No. | Retention time (min) | Identification | Formula | Adduct | Extract mass | Delta (ppm) | Matching rate | Trend | *p* value | HMDB ID | KEGG ID | Involved Pathway |
| --- | --- | --- | --- | --- | --- | --- | --- | --- | --- | --- | --- | --- | --- |
| Positive | 1 | 1.56 | L-Isoleucine (leucine) | C6H13NO2 | [M+H]+ | 132.1018 | 1 | 97.0% | ↑ | 5.1266E-6 | HMDB0000172  (HMDB0000687) | C00407  (C00123) | B, C, F |
|  | 2 | 1.60 | Propionylcarnitine | C10H19NO4 | [M+H]+ | 218.1387 | 1 | 100.0% | ↑ | 0.02415 | HMDB0000824 | C03017 | - |
|  | 3 | 3.24 | L-Phenylalanine | C9H11NO2 | [M+H]+ | 166.0861 | 1 | 100.0% | ↑ | 0.00699 | HMDB0000159 | C00079 | B, E, G |
|  | 4 | 4.04 | Pantothenic acid | C9H17NO5 | [M+H]+ | 220.1181 | 1 | 80.5% | ↑ | 1.0572E-5 | HMDB0000210 | C00864 | H |
|  | 5 | 4.68 | Isovalerylcarnitine | C12H23NO4 | [M+H]+ | 246.1698 | 1 | 100.0% | ↑ | 2.0204E-5 | HMDB0000688 | NA | - |
|  | 6 | 8.00 | Phytosphingosine | C18H39NO3 | [M+H]+ | 318.3006 | 4 | 100.0% | ↓ | 0.03233 | HMDB0004610 | C12144 | A |
|  | 7 | 8.45 | Sphinganine | C18H39NO2 | [M+H]+ | 302.3056 | 4 | 100.0% | ↓ | 4.9255E-6 | HMDB0000269 | C00836 | A |
|  | 8 | 8.80 | Eplerenone | C24H30O6 | [M+H]+ | 415.2116 | 2 | 100.0% | ↓ | 0.02025 | HMDB0014838 | C12512 | - |
|  | 9 | 9.42 | 2-methoxyestrone | C19H24O3 | [M+H]+ | 301.1799 | 6 | 93.4% | ↓ | 0.00474 | HMDB0000010 | C05299 | O |
|  | 10 | 11.68 | 11-octadecenylcarnitine | C_25_H_47_NO_4_ | [M+H]^+^ | 426.3577 | 3 | 100.0% | ↑ | 0.00120 | HMDB0013338 | NA | - |
|  | 11 | 14.74 | LysoPC (20:5) | C28H48NO7P | [M+H]+ | 542.3232 | 4 | 100.0% | ↓ | 0.04161 | HMDB0010397 | C04230 | I |
|  | 12 | 14.80 | LysoPE (20:4/0:0) | C25H44NO7P | [M+H]+ | 502.2926 | 2 | 100.0% | ↓ | 0.02867 | HMDB0011518 | NA | - |
| Negative | 4 | 3.82 | Pantothenic acid | C9H17NO5 | [M-H]- | 218.1044 | 4 | 97.3% | ↑ | 0.00046 | HMDB0000210 | C00864 | H |
|  | 13 | 4.44 | L-Tryptophan | C11H12N2O2 | [M-H]- | 203.0834 | 4 | 100.0% | ↑ | 0.00252 | HMDB0000929 | C00078 | B, M |
|  | 14 | 12.28 | LysoPC (14:0/0:0) | C22H46NO7P | [M+FA-H]- | 512.3023 | 6 | 94.9% | ↓ | 0.00145 | HMDB0010379 | C04230 | I |
|  | 15 | 13.07 | Sphingosine-1-phosphate (S1P) | C18H38NO5P | [M-H]- | 378.2428 | 6 | 100.0% | ↑ | 0.03737 | HMDB0000277 | C06124 | A |
|  | 16 | 13.10 | LysoPC (16:1) | C24H48NO7P | [M+FA-H]- | 538.3152 | 7 | 94.6% | ↓ | 0.00114 | HMDB0010383 | C04230 | I |
|  | 17 | 14.24 | LysoPE (0:0/20:2) | C25H48NO7P | [M-H]- | 504.3099 | 0 | 100.0% | ↓ | 0.00341 | HMDB0011483 | NA | - |
|  | 18 | 14.28 | LysoPC (22:6) | C30H50NO7P | [M+FA-H]- | 612.3323 | 5 | 93.8% | ↓ | 0.00862 | HMDB0010404 | C04230 | I |
|  | 19 | 14.43 | LysoPE (0:0/22:4) | C27H48NO7P | [M-H]- | 528.3099 | 4 | 100.0% | ↓ | 0.00082 | HMDB0011493 | NA | - |
|  | 20 | 14.61 | LysoPC (20:4) | C28H50NO7P | [M+FA-H]- | 588.3321 | 7 | 94.4% | ↓ | 0.00181 | HMDB0010395 | C04230 | I |
|  | 21 | 14.74 | LysoPC (18:2) | C26H50NO7P | [M+FA-H]- | 564.3314 | 1 | 90.8% | ↓ | 0.00286 | HMDB0010386 | C04230 | I |
|  | 22 | 16.59 | LysoPC (16:0) | C24H50NO7P | [M+FA-H]- | 540.3317 | 8 | 94.9% | ↓ | 0.00827 | HMDB0010382 | C04230 | I |
|  | 23 | 16.63 | LysoPC (15:0) | C23H48NO7P | [M-H]- | 480.3106 | 7 | 100.0% | ↓ | 0.01072 | HMDB0010381 | C04230 | II |
|  | 24 | 17.54 | LysoPC (18:1) | C26H52NO7P | [M+FA-H]- | 566.3473 | 6 | 95.0% | ↓ | 0.00103 | HMDB0002815 | C04230 | I |
|  | 25 | 17.56 | LysoPE (0:0/20:1) | C25H50NO7P | [M-H]- | 506.3256 | 5 | 100.0% | ↓ | 0.00135 | HMDB0011482 | NA | - |
|  | 26 | 18.01 | Eicosapentaenoic acid | C20H30O2 | [M-H]- | 301.2181 | 5 | 93.5% | ↓ | 0.00864 | HMDB0001999 | C06428 | D |
|  | 27 | 18.28 | Punicic acid | C18H30O2 | [M-H]- | 277.2173 | 5 | 92.8% | ↓ | 0.00150 | HMDB0030963 | C08364 | - |
|  | 28 | 19.65 | LysoPC (20:2) | C28H54NO7P | [M+FA-H]- | 592.3617 | 7 | 90.0% | ↓ | 0.01320 | HMDB0010392 | C04230 | I |
|  | 29 | 21.21 | Arachidonic acid | C20H32O2 | [M-H]- | 303.2336 | 6 | 100.0% | ↑ | 0.00109 | HMDB0060102 | C00219 | D, J |
|  | 30 | 22.54 | LysoPC (17:0) | C25H52NO7P | [M-H]- | 508.3433 | 6 | 88.7% | ↓ | 0.00316 | HMDB0012108 | C04230 | I |
|  | 31 | 24.33 | Palmitic acid | C16H32O2 | [M-H]- | 255.2338 | 5 | 100.0% | ↓ | 0.00108 | HMDB0000220 | C00249 | D, K, L, N |

Ⅰ) Compared with control group, content increased ↑; content decreased ↓.

II) Pathway: (A) Sphingolipid metabolism; (B) Aminoacyl-tRNA biosynthesis;; (C) Valine, leucine and isoleucine biosynthesis; (D) Biosynthesis of unsaturated fatty acids; (E) Phenylalanine, tyrosine and tryptophan biosynthesis; (F)Valine, leucine and isoleucine degradation; (G) Phenylalanine metabolism; (H) Pantothenate and CoA biosynthesis; (I) Glycerophospholipid metabolism; (J) Arachidonic acid metabolism; (K) Fatty acid elongation; (L) Fatty acid degradation; (M) Tryptophan metabolism; (N) Fatty acid biosynthesis; (O) Steroid hormone biosynthesis.

III) “-” stands for no hits in the database.

**Supplementary Table 3.**Differential endogenous metabolites in the plasma from rats after administration and their identification results.

| Ion mode | No. | Retention time (min) | Identification | Formula | Adduct | Extract mass | Delta (ppm) | Matching rate | Trend | *p* value | HMDB ID | KEGG ID | Involved Pathway |
| --- | --- | --- | --- | --- | --- | --- | --- | --- | --- | --- | --- | --- | --- |
| Positive | 1^#^ | 1.56 | L-Isoleucine (leucine) | C_6_H_13_NO_2_ | [M+H]^+^ | 132.1018 | 1 | 97.0% | ↓ | 3.0062E-5 | HMDB0000172  (HMDB0000687) | C00407  (C00123) | A, C, D |
|  | 2^#^ | 3.24 | L-Phenylalanine | C_9_H_11_NO_2_ | [M+H]^+^ | 166.0861 | 1 | 100.0% | ↓ | 3.2485E-6 | HMDB0000159 | C00079 | A, E, F |
|  | 3^#^ | 4.68 | Isovalerylcarnitine | C_12_H_23_NO_4_ | [M+H]^+^ | 246.1698 | 1 | 100.0% | ↓ | 4.9130E-9 | HMDB0000688 | NA | - |
|  | 4^#^ | 8.00 | Phytosphingosine | C_18_H_39_NO_3_ | [M+H]^+^ | 318.3006 | 4 | 100.0% | ↑ | 0.02048 | HMDB0004610 | C12144 | B, |
|  | 5^#^ | 8.45 | Sphinganine | C_18_H_39_NO_2_ | [M+H]^+^ | 302.3056 | 4 | 100.0% | ↑ | 0.01024 | HMDB0000269 | C00836 | B, |
|  | 6 | 9.40 | *β*-Costic acid | C_15_H_22_O_2_ | [M+H]^+^ | 235.1693 | 2 | 100.0% | ↑ | 0.03971 | HMDB0035794 | NA | - |
|  | 7^#^ | 11.68 | 11-octadecenylcarnitine | C_25_H_47_NO_4_ | [M+H]^+^ | 426.3577 | 3 | 100.0% | ↑ | 0.04798 | HMDB0013338 | NA | - |
|  | 8^#^ | 14.74 | LysoPC (20:5) | C_28_H_48_NO_7_P | [M+H]^+^ | 542.3232 | 4 | 100.0% | ↑ | 0.02418 | HMDB0010397 | C04230 | H |
|  | 9 | 15.48 | LysoPC (18:2) | C_26_H_50_NO_7_P | [M+H]^+^ | 520.3393 | 7 | 100.0% | ↑ | 0.00490 | HMDB0010386 | C04230 | H |
|  | 10 | 16.59 | LysoPC (16:0) | C_24_H_50_NO_7_P | [M+H]^+^ | 496.3389 | 1 | 98.3% | ↑ | 0.02329 | HMDB0010382 | C04230 | H |
| Negative | 9^#^ | 14.74 | LysoPC (18:2) | C_26_H_50_NO_7_P | [M+FA-H]^-^ | 564.3314 | 1 | 90.8% | ↑ | 0.00116 | HMDB0010386 | C04230 | H |
|  | 10^#^ | 16.59 | LysoPC (16:0) | C_24_H_50_NO_7_P | [M+FA-H]^-^ | 540.3317 | 8 | 94.9% | ↑ | 2.8384E-6 | HMDB0010382 | C04230 | H |
|  | 11^#^ | 4.44 | L-Tryptophan | C_11_H_12_N_2_O_2_ | [M-H]^-^ | 203.0834 | 4 | 100.0% | ↓ | 4.1109E-11 | HMDB0000929 | C00078 | A, I |
|  | 12^#^ | 13.07 | Sphingosine-1-phosphate (S1P) | C_18_H_38_NO_5_P | [M-H]^-^ | 378.2428 | 6 | 100.0% | ↓ | 0.01981 | HMDB0000277 | C06124 | B, |
|  | 13 | 13.10 | Lysopc(16:1) | C_24_H_48_NO_7_P | [M+FA-H]^-^ | 538.3152 | 7 | 94.6% | ↑ | 0.00010 | HMDB0010383 | C04230 | I |
|  | 14^#^ | 14.24 | LysoPE (0:0/20:2) | C_25_H_48_NO_7_P | [M-H]^-^ | 504.3099 | 0 | 100.0% | ↑ | 0.00064 | HMDB0011483 | NA | - |
|  | 15^#^ | 14.43 | LysoPE (0:0/22:4) | C_27_H_48_NO_7_P | [M-H]^-^ | 528.3099 | 4 | 100.0% | ↑ | 5.8403E-5 | HMDB0011493 | NA | - |
|  | 16^#^ | 14.61 | LysoPC (20:4) | C_28_H_50_NO_7_P | [M+FA-H]^-^ | 588.3321 | 7 | 94.4% | ↑ | 1.5134E-5 | HMDB0010395 | C04230 | H |
|  | 17 | 14.63 | LysoPE (0:0/22:6) | C_27_H_44_NO_7_P | [M-H]^-^ | 524.2791 | 6 | 100.0% | ↑ | 0.02342 | HMDB0011496 | NA | - |
|  | 18^#^ | 16.63 | LysoPC (15:0) | C_23_H_48_NO_7_P | [M-H]^-^ | 480.3106 | 7 | 100.0% | ↑ | 5.0580E-7 | HMDB0010381 | C04230 | H |
|  | 19^#^ | 17.54 | LysoPC (18:1) | C_26_H_52_NO_7_P | [M+FA-H]^-^ | 566.3473 | 6 | 95.0% | ↑ | 2.1776E-5 | HMDB0002815 | C04230 | H |
|  | 20^#^ | 17.56 | LysoPE (0:0/20:1) | C_25_H_50_NO_7_P | [M-H]^-^ | 506.3256 | 5 | 100.0% | ↑ | 0.00135 | HMDB0011482 | NA | - |
|  | 21^#^ | 18.01 | Eicosapentaenoic acid | C_20_H_30_O_2_ | [M-H]^-^ | 301.2181 | 5 | 93.5% | ↑ | 0.01282 | HMDB0001999 | C06428 | G |

Ⅰ) Compared with model group, content increased ↑; content decreased ↓.

II) Pathway: (A) Aminoacyl-tRNA biosynthesis; (B) Sphingolipid metabolism; (C) Valine, leucine and isoleucine biosynthesis; (D) Valine, leucine and isoleucine degradation; (E) Phenylalanine, tyrosine and tryptophan biosynthesis; (F) Phenylalanine metabolism; (G) Biosynthesis of unsaturated fatty acids; (H) Glycerophospholipid metabolism; (I) Tryptophan metabolism.

III) “-” stands for no hits in the database.

IV) “#” stands for the endogenous metabolites reported in Table S1.

**Supplementary Table 4.**Identification of prototypes and metabolites from prepared rhubarb in rats plasma.

| Ion mode | No. | Retention time (min) | Identified compounds | Formula | TOF-MS | | TOF-MS/MS | | Identification | |
| --- | --- | --- | --- | --- | --- | --- | --- | --- | --- | --- |
|  |  |  |  |  | Detected mass | Mass error (ppm) | Cracking fragments (elemental composition) | Purity score | Prototype | Metabolic way |
| Negative | 1 | 9.13 | Rhein (P1) | C_15_H_8_O_6_ | 283.0251 [M-H]^-^ | 1.0 | 239[M-H-CO_2_]^-^, 211[M-H-CO_2_-CO]^-^, 183[M-H-CO_2_-2CO]^-^ | 80.8% | － | － |
|  | 2 | 10.70 | Emodin (P2) | C_15_H_10_O_5_ | 269.0457 [M-H]^-^ | 0.7 | 241[M-H-CO]^-^, 225[M-H-CO_2_]^-^ | 96.2% | － | － |
|  | 3 | 8.15 | Aloe-emodin (P3) | C_15_H_10_O_5_ | 269.0459 [M-H]^-^ | 1.3 | 240[M-H-CHO]-,211[M-H-2CHO]^-^, 167[M-H-2CHO-CO_2_]^-^ | 96.2% | － | － |
|  | 4 | 8.612 | Chrysophanol (P4) | C_15_H_10_O_4_ | 253.0509 [M-H]^-^ | 0.9 | 225[M-H-CO]^-^,210[M-H-CO-CH_3_]^-^, 182[M-H-2CO-CH_3_]^-^ | 81.5% | － | － |
|  | 5 | 8.61 | 6-methyl-rhein (P5) | C_16_H_10_O_6_ | 297.0407 [M-H]^-^ | 0.8 | 253[M- H-CO_2_]^-^, 225[M-H-C_2_H_4_-CO_2_]^-^ | 92.2% | － | － |
|  | 6 | 8.62 | Emodin-1(6)-*O*-β-D-glucopyranoside (P6) | C_21_H_20_O_10_ | 431.0988 [M-H]^-^ | 1.0 | 255[M-H-C_6_H_8_O_6_]^-^, 175[C_6_H_8_O_6_]^-^ | 82.5% | － | － |
|  | 7 | 6.62 | Isorhapontin (P7) | C_21_H_24_O_9_ | 419.1347 [M-H]^-^ | -0.2 | 243[M-H-C_6_H_12_O_5_]^-^, 175[M-C_6_H_11_O_6_-C_4_H_2_-OH]^-^ | 100.0% | － | － |
|  | 8 | 6.48 | 2,5-dimethyl-7-hydroxychromone (P8) | C_11_H_10_O_3_ | 189.0558 [M-H]^-^ | 0.4 | 161[M-H-CO]^-^,147[M-H-CH_2_-CO]^-^, 133[M-H-CH_3_-CH_2_O]^-^ | 89.9% | － | － |
|  | 9 | 8.63 | 3,4',5-trihydroxystlbene-4'-*O*-β-D-glucopyranoside(P9) | C_20_H_22_O_8_ | 389.1313 [M-H]^-^ | 1.0 | 213[M-H-C_6_H_8_O_6_]^-^, 175[C_6_H_8_O_6_]^-^ | 82.5% | － | － |
|  | 10 | 7.28 | Hydroxy-rhein monosulfate (M1) | C_15_H_8_O_10_S | 378.9769 [M-H]^-^ | 0.9 | 299[M-H-SO_3_]^-^,255[M-H-CO_2_-SO_3_]^-^, 227[M-H-C_2_H_4_-CO_2_-SO_3_]^-^ | 100.0% | Rhein | Hydroxylation+Sulfation |
|  | 11 | 7.00 | Rhein monosulfate (M2) | C_15_H_8_O_9_S | 362.9818 [M-H]^-^ | 0.5 | 283[M-H-SO_3_]^-^,239[M-H-CO_2_-SO_3_]^-^, 211[M-H-C_2_H_4_-CO_2_-SO_3_]^-^ | 92.3% | Rhein | Sulfation |
|  | 12 | 6.65 | Rhein glucuronide (M3) | C_21_H_16_O_12_ | 459.0564 [M-H]^-^ | -1.2 | 283[M-H-C_6_H_8_O_6_]^-^, 239[M-H-C_6_H_12_O_6_-CO_2_]^-^ | 100.0% | Rhein | Glucuronidation |
|  | 13 | 8.15 | Rhein-9-anthrone (M4) | C_15_H_10_O_5_ | 269.0457 [M-H]^-^ | 0.7 | 241[M-H-CO]^-^, 225[M-H-CO_2_]^-^ | 91.7% | Sennoside A /B | Hydrolyzation |
|  | 14 | 8.64 | Methyl-Rhein (M5) | C_16_H_10_O_6_ | 297.0407 [M-H]^-^ | 0.8 | 225[M-H-C_3_H_3_O_2_]^-^, 253[M-H-CO_2_]^-^ | 100.0% | Rhein | Methylation |
|  | 15 | 9.15 | Oxidative -Rhein(M6) | C_15_H_8_O_7_ | 299.0196 [M-H]^-^ | -0.4 | 227[M-H-C_3_H_2_O_2_]^-^, 255[M-H- CO_2_]^-^ | 75.7% | Rhein | Oxidation |
|  | 16 | 8.62 | Hydroxy-Rhein (M7) | C_15_H_10_O_4_ | 253.0509 [M-H]^-^ | 0.9 | 205[M-H-CH_2_O_2_]^-^,225[M-H-CO]^-^ | 77.3% | Rhein | Hydroxylation |
|  | 17 | 6.80 | Hydroxy-emodin monosulfate (M8) | C_15_H_10_O_9_S | 364.9968 [M-H]^-^ | -1.2 | 285[M-H-SO_3_]^-^, 250[M-2H-SO_4_-OH]^-^ | 100.0% | Emodin | Hydroxylation+Sulfation |
|  | 18 | 9.14 | Rhein decarbonylate (M9) | C_14_H_8_O_5_ | 255.0301 [M-H]^-^ | 0.7 | 255[M-H]- ,227[M-H-CO]^-^, 211[M-H-CO_2_]^-^ | 87.1% | Rhein | Decarbonylation |
|  | 19 | 9.08 | 1, 8-dihydroxy-anthraquinone (M10) | C_14_H_8_O_4_ | 239.0351 [M-H]^-^ | 0.5 | 239[M-H]^-^, 223[M-H-OH]^-^ | 87.5% | Rhein | Decarboxylation |
|  | 20 | 6.91 | Hydroxy-emodin glucuronide (M11) | C_21_H_18_O_12_ | 461.0722 [M-H]^-^ | -0.7 | 461[M-H]^-^, 285[M-H-C_6_H_8_O_6_]^-^ | 100.0% | Emodin | Hydroxylation+Glucuronidation |
|  | 21 | 6.90 | Aloe-emodin monosulfate (M12) | C_15_H_10_O_8_S | 349.0027 [M-H]^-^ | 0.8 | 269[M-H-SO_3_H]^-^ | 93.2% | Aloe-emodin | Sulfation |
|  | 22 | 8.15 | Aloe-emodin Glucuronide(M13) | C_21_H_18_O_11_ | 445.0778 [M-H]^-^ | 0.4 | 269[M-H-C_6_H_8_O_6_]^-^ | 100.0% | Aloe-emodin | Glucuronidation |
|  | 23 | 7.92 | Chrysophanol monosulfate (M14) | C_15_H_10_O_7_S | 333.0075 [M-H]^-^ | 0.2 | 253[M-H-SO_3_H]^-^ | 100.0% | Chrysophanol | Sulfation |
|  | 24 | 7.73 | Emodin-1-*O*-β-D-glucopyranoside -Desaturation (M15) | C_21_H_18_O_10_ | 429.0833 [M-H]^-^ | 1.3 | 253[M-H-C_6_H_8_O_6_]^-^ | 93.2% | Emodin-1(6)-*O*-β-D-glucopyranoside | Glucopyranoside Desaturation |
|  | 25 | 6.76 | Emodin-1-*O*-β-D-glucopyranoside - glucuronide (M16) | C_27_H_28_O_15_ | 591.1355 [M-H]^-^ | -0.1 | 429[M-H-C_6_H_9_O_5_]^-^,253[M-H-C_12_H_12_O_11_]^-^ | 88.9% | Emodin-1(6)-*O*-β-D-glucopyranoside | Glucuronidation |
|  | 26 | 7.71 | Methyl-Emodin-1-*O*-β-D-glucopyranoside (M17) | C_22_H_22_O_10_ | 429.0827 [M+FA-H]^-^ | -0.1 | 253[M-H-OCH_3_-C_6_H_11_O_5_]^-^ | 100.0% | Emodin-1(6)-*O*-β-D-glucopyranoside | Methylation |
| Positive | 1 | 9.61 | Rhein (P1) | C_15_H_8_O_6_ | 285.0398 [M+H]^+^ | 1.5 | 267[M+H-OH]^+^,253[M+H-2OH]+,241[M+H-COOH]^+^,  225[M+H-OH-COOH]^+^ | 85.2% | － | － |
|  | 2 | 7.93 | Chrysophan(P4) | C_15_H_10_O_4_ | 255.0653 [M+H]^+^ | 0.6 | 237[M+H-OH]^+^, 227[M+H-CO]^+^, 149[C_8_H_5_O_3_]^+^ | 78.0% | － | － |
|  | 3 | 8.35 | Aloe-emodin(P3) | C_15_H_10_O_5_ | 271.0603 [M+H]^+^ | 0.7 | 253[M+H-OH]^+^, 241[M+H-CH-OH]^+^, 225[M+H-OH-CHOH]^+^ | 85.5% | － | － |
|  | 4 | 8.82 | 6-methyl-rhein(P5) | C_16_H_10_O_6_ | 299.0556 [M+H]^+^ | 1.8 | 283[M+H-OH]^+^, 267[M+H-OH-CH3]^+^, 253[M+H-COOH]^+^ | 86.8% | － | － |
|  | 5 | 7.11 | Hydrogen-Rhein (M18) | C_15_H_10_O_6_ | 287.0551 [M+H]^+^ | 0.3 | 269[M+H-OH]^+^, 251[M+H-2OH]^+^,  241[M+H-CH_3_O_2_]^+^, 233[M+H-OH-CH_3_O_2_]^+^ | 80.6% | Rhein | Hydrogenation |
|  | 6 | 8.38 | Emodin- glucuronide (M19) | C_21_H_18_O_11_ | 447.0926 [M+H]^+^ | 0.9 | 271[M+H-C_6_H_9_O_6_]^+^ | 100.0% | Emodin | Glucuronidation |

**Supplementary Table 5.**Gray correlation grade with rank order of 28 drug relative compounds detected in rat plasma.

| Compounds | Average gray correlation grade | Order |
| --- | --- | --- |
|  |  |  |
| Rhein | 0.7030 | 10 |
| Emodin | 0.6628 | 28 |
| Aloe-emodin | 0.6868 | 23 |
| Chrysophanol | 0.6985 | 15 |
| 6-methyl-rhein | 0.6752 | 24 |
| Emodin-1-*O*-β-D-glucopyranoside/ Emodin-6-*O*-β-D-glucopyranoside | 0.6938 | 17 |
| Isorhapontin | 0.6999 | 13 |
| 2,5-dimethyl-7-hydroxychromone | 0.7027 | 11 |
| 3,4',5-trihydroxystlbene-4'-*O*-β-D-glucopyranoside | 0.7014 | 12 |
| Hydroxy-rhein monosulfate (M1) | 0.7271 | 7 |
| Rhein monosulfate (M2) | 0.6908 | 19 |
| Rhein glucuronide (M3) | 0.7359 | 3 |
| Rhein-9-anthrone (M4) | 0.6879 | 21 |
| Methyl-Rhein (M5) | 0.6730 | 26 |
| Oxidative -Rhein (M6) | 0.7324 | 4 |
| Hydroxy-Rhein (M7) | 0.6935 | 18 |
| Hydroxy-emodin monosulfate (M8) | 0.7200 | 8 |
| Rhein decarbonylate (M9) | 0.6995 | 14 |
| 1, 8-dihydroxy-anthraquinone (M10) | 0.7299 | 5 |
| Hydroxy-emodin glucuronide (M11) | 0.7463 | 2 |
| Aloe-emodin monosulfate (M12) | 0.6876 | 22 |
| Aloe-emodin glucuronide (M13) | 0.7282 | 6 |
| Chrysophanol monosulfate (M14) | 0.7552 | 1 |
| Emodin-1-*O*-β-D-glucopyranoside -Desaturation (M15) | 0.7123 | 9 |
| Emodin-1-*O*-β-D-glucopyranoside - glucuronide (M16) | 0.6972 | 16 |
| Methyl-Emodin-1-*O*-β-D-glucopyranoside (M17) | 0.6884 | 20 |
| Hydrogen-Rhein (M18) | 0.6743 | 25 |
| Emodin- glucuronide (M19) | 0.6700 | 27 |

**Supplementary Table 6.**Gray correlation grade with rank order of 28 drug relative compounds detected in rat plasma in detail.

| Compounds | Gray correlation grade | | | | | | | | | | | | | | | | | |
| --- | --- | --- | --- | --- | --- | --- | --- | --- | --- | --- | --- | --- | --- | --- | --- | --- | --- | --- |
|  | L-isoleucine (leucine) | L-phenylalanine | Phytosphingosine | Sphinganine | S1P | L-tryptophan | LysoPC  (16:0) | LysoPC  (20:4) | LysoPC  (18:1) | LysoPC  (15:0) | Eicosapentaenoic acid | Isovalerylcarnitine | LysoPC (20:5) | 11-octadecenylcarnitine | LysoPC  (18:2) | LysoPE  (0:0/20:2) | LysoPE  (0:0/22:4) | LysoPE  (0:0/20:1) |
| Rhein | 0.7086 | 0.6733 | 0.7487 | 0.7567 | 0.6569 | 0.6404 | 0.6382 | 0.7629 | 0.7774 | 0.6556 | 0.6996 | 0.7851 | 0.6459 | 0.6906 | 0.7575 | 0.7034 | 0.6934 | 0.6594 |
| Emodin | 0.7161 | 0.7009 | 0.7375 | 0.7120 | 0.7386 | 0.6404 | 0.6480 | 0.5766 | 0.6126 | 0.6612 | 0.5633 | 0.6540 | 0.7052 | 0.5709 | 0.6540 | 0.6660 | 0.6881 | 0.6851 |
| Aloe-emodin | 0.6842 | 0.7515 | 0.6880 | 0.7631 | 0.7128 | 0.6862 | 0.7252 | 0.6684 | 0.7237 | 0.6999 | 0.6163 | 0.6885 | 0.6183 | 0.6219 | 0.7263 | 0.7223 | 0.6254 | 0.6410 |
| Chrysophanol | 0.6943 | 0.6629 | 0.8006 | 0.7743 | 0.6816 | 0.6842 | 0.6702 | 0.7080 | 0.7355 | 0.6743 | 0.6548 | 0.6401 | 0.6565 | 0.6258 | 0.7578 | 0.7784 | 0.7000 | 0.6738 |
| 6-methyl-rhein | 0.6205 | 0.6640 | 0.6817 | 0.7280 | 0.6436 | 0.6760 | 0.6828 | 0.6743 | 0.7184 | 0.6906 | 0.6279 | 0.6729 | 0.6906 | 0.6737 | 0.7073 | 0.7121 | 0.6623 | 0.6274 |
| Emodin-1-*O-*β-D-glucopyranoside  /Emodin-6-*O*-β-D-glucopyranoside | 0.7325 | 0.6571 | 0.7254 | 0.6305 | 0.8165 | 0.5918 | 0.6962 | 0.7148 | 0.6874 | 0.6317 | 0.6830 | 0.8322 | 0.7171 | 0.7209 | 0.6600 | 0.6701 | 0.7267 | 0.5943 |
| Isorhapontin | 0.6626 | 0.6873 | 0.6480 | 0.7314 | 0.6640 | 0.7107 | 0.7020 | 0.6655 | 0.8198 | 0.7094 | 0.6092 | 0.6656 | 0.6496 | 0.8291 | 0.6838 | 0.7830 | 0.6439 | 0.7325 |
| 2,5-dimethyl-7-hydroxychromone | 0.6636 | 0.6550 | 0.7355 | 0.7686 | 0.7437 | 0.6708 | 0.6889 | 0.6942 | 0.7141 | 0.6904 | 0.6600 | 0.7480 | 0.6679 | 0.5594 | 0.7169 | 0.6600 | 0.8002 | 0.8112 |
| 3,4',5-trihydroxystlbene-4'-*O*-β-D-glucopyranoside | 0.6373 | 0.7528 | 0.7820 | 0.7469 | 0.6342 | 0.7396 | 0.7091 | 0.6689 | 0.7449 | 0.6550 | 0.7053 | 0.6757 | 0.63 | 0.579 | 0.7331 | 0.7344 | 0.7824 | 0.7151 |
| Hydroxy-rhein monosulfate (M1) | 0.6793 | 0.7156 | 0.7957 | 0.7711 | 0.6678 | 0.6927 | 0.7416 | 0.7561 | 0.9724 | 0.7255 | 0.5907 | 0.6654 | 0.7747 | 0.612 | 0.7686 | 0.7372 | 0.6541 | 0.7666 |
| Rhein monosulfate (M2) | 0.6599 | 0.6697 | 0.6581 | 0.6725 | 0.6912 | 0.6808 | 0.7850 | 0.6725 | 0.6552 | 0.7629 | 0.6846 | 0.7269 | 0.6852 | 0.6605 | 0.6594 | 0.6862 | 0.7162 | 0.7074 |
| Rhein glucuronide (M3) | 0.6880 | 0.6765 | 0.7438 | 0.7587 | 0.6663 | 0.6983 | 0.6790 | 0.7560 | 0.8206 | 0.6867 | 0.8240 | 0.6531 | 0.7297 | 0.8456 | 0.8252 | 0.8129 | 0.6824 | 0.6989 |
| Rhein-9-anthrone (M4) | 0.6517 | 0.6838 | 0.7072 | 0.7435 | 0.7273 | 0.6337 | 0.6874 | 0.6751 | 0.6969 | 0.7041 | 0.6433 | 0.7354 | 0.6725 | 0.5643 | 0.7094 | 0.6555 | 0.6934 | 0.7974 |
| Methyl-Rhein (M5) | 0.7049 | 0.6630 | 0.7043 | 0.7062 | 0.6196 | 0.6822 | 0.6825 | 0.6766 | 0.6725 | 0.6727 | 0.6037 | 0.6547 | 0.735 | 0.7109 | 0.6606 | 0.6642 | 0.6355 | 0.6657 |
| Oxidative -Rhein (M6) | 0.7133 | 0.7006 | 0.7393 | 0.7379 | 0.7149 | 0.7202 | 0.8216 | 0.6953 | 0.7455 | 0.8750 | 0.7823 | 0.6981 | 0.6887 | 0.6481 | 0.7629 | 0.7066 | 0.7073 | 0.7250 |
| Hydroxy-Rhein (M7) | 0.6672 | 0.6967 | 0.6955 | 0.6808 | 0.6898 | 0.7181 | 0.7039 | 0.6846 | 0.7302 | 0.7120 | 0.6655 | 0.7054 | 0.6434 | 0.6591 | 0.7438 | 0.7531 | 0.6844 | 0.6493 |
| Hydroxy-emodin monosulfate (M8) | 0.7156 | 0.6873 | 0.6999 | 0.7192 | 0.7089 | 0.7010 | 0.7503 | 0.7135 | 0.7599 | 0.7491 | 0.6939 | 0.7209 | 0.6791 | 0.7706 | 0.7404 | 0.6974 | 0.7214 | 0.7312 |
| Rhein decarbonylate (M9) | 0.7540 | 0.7507 | 0.7970 | 0.6627 | 0.7290 | 0.7064 | 0.7319 | 0.7695 | 0.6333 | 0.7484 | 0.6645 | 0.6444 | 0.6963 | 0.6075 | 0.6733 | 0.6802 | 0.6572 | 0.6844 |
| 1, 8-dihydroxy-anthraquinone (M10) | 0.7655 | 0.7783 | 0.7035 | 0.7269 | 0.7629 | 0.7922 | 0.6763 | 0.6914 | 0.7134 | 0.6869 | 0.7861 | 0.6878 | 0.7424 | 0.7457 | 0.7224 | 0.7111 | 0.7267 | 0.7184 |
| Hydroxy-emodin glucuronide (M11) | 0.6849 | 0.7082 | 0.7603 | 0.7076 | 0.7146 | 0.7307 | 0.6699 | 0.7170 | 0.7989 | 0.6916 | 0.7882 | 0.7198 | 0.7401 | 0.7323 | 0.8102 | 0.7883 | 0.8282 | 0.8422 |
| Aloe-emodin monosulfate (M12) | 0.6710 | 0.6750 | 0.6641 | 0.6521 | 0.6639 | 0.6858 | 0.7872 | 0.7098 | 0.6543 | 0.7601 | 0.7611 | 0.6973 | 0.6835 | 0.7017 | 0.6400 | 0.6465 | 0.6663 | 0.6568 |
| Aloe-emodin glucuronide (M13) | 0.6943 | 0.7269 | 0.7213 | 0.7212 | 0.7179 | 0.7656 | 0.7923 | 0.7833 | 0.7089 | 0.7848 | 0.6491 | 0.7562 | 0.7708 | 0.8193 | 0.6911 | 0.6786 | 0.6817 | 0.6447 |
| Chrysophanol monosulfate (M14) | 0.6952 | 0.6878 | 0.7917 | 0.7830 | 0.6741 | 0.7036 | 0.7885 | 0.7236 | 0.8249 | 0.7802 | 0.7108 | 0.7180 | 0.7228 | 0.7826 | 0.8175 | 0.7915 | 0.7896 | 0.8084 |
| Emodin-1-*O*-β-D-glucopyranoside-Desaturation (M15) | 0.7203 | 0.7480 | 0.7029 | 0.6747 | 0.7254 | 0.7736 | 0.6927 | 0.7775 | 0.7759 | 0.6955 | 0.6303 | 0.6580 | 0.7205 | 0.7258 | 0.7239 | 0.6934 | 0.6763 | 0.7071 |
| Emodin-1-*O*-β-D-glucopyranoside- glucuronide (M16) | 0.7459 | 0.8015 | 0.7149 | 0.6708 | 0.7831 | 0.7674 | 0.7025 | 0.6715 | 0.6710 | 0.7092 | 0.7020 | 0.7569 | 0.737 | 0.5692 | 0.6659 | 0.6770 | 0.6110 | 0.5923 |
| Methyl-Emodin-1-*O*-β-D-glucopyranoside (M17) | 0.7062 | 0.6138 | 0.6287 | 0.5981 | 0.7587 | 0.5628 | 0.6578 | 0.7337 | 0.7773 | 0.5967 | 0.7822 | 0.7844 | 0.7302 | 0.6043 | 0.7267 | 0.6419 | 0.6528 | 0.8357 |
| Hydrogen-Rhein (M18) | 0.6967 | 0.6972 | 0.6680 | 0.6731 | 0.6463 | 0.6666 | 0.7603 | 0.9306 | 0.6024 | 0.6789 | 0.6265 | 0.5903 | 0.6162 | 0.6419 | 0.5535 | 0.6700 | 0.7497 | 0.6694 |
| Emodin- glucuronide (M19) | 0.5441 | 0.7099 | 0.6868 | 0.6378 | 0.6295 | 0.6403 | 0.7904 | 0.6533 | 0.6889 | 0.7657 | 0.6542 | 0.6112 | 0.8315 | 0.7809 | 0.6482 | 0.6090 | 0.5734 | 0.6041 |


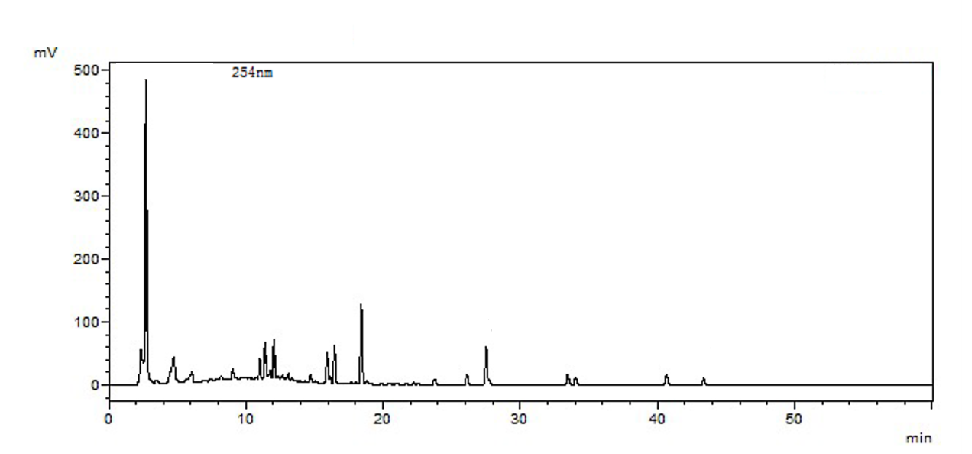
**Supplementary Figure 1.** Identical HPLC chromatogram of prepared rhubarb for chemoprofile.


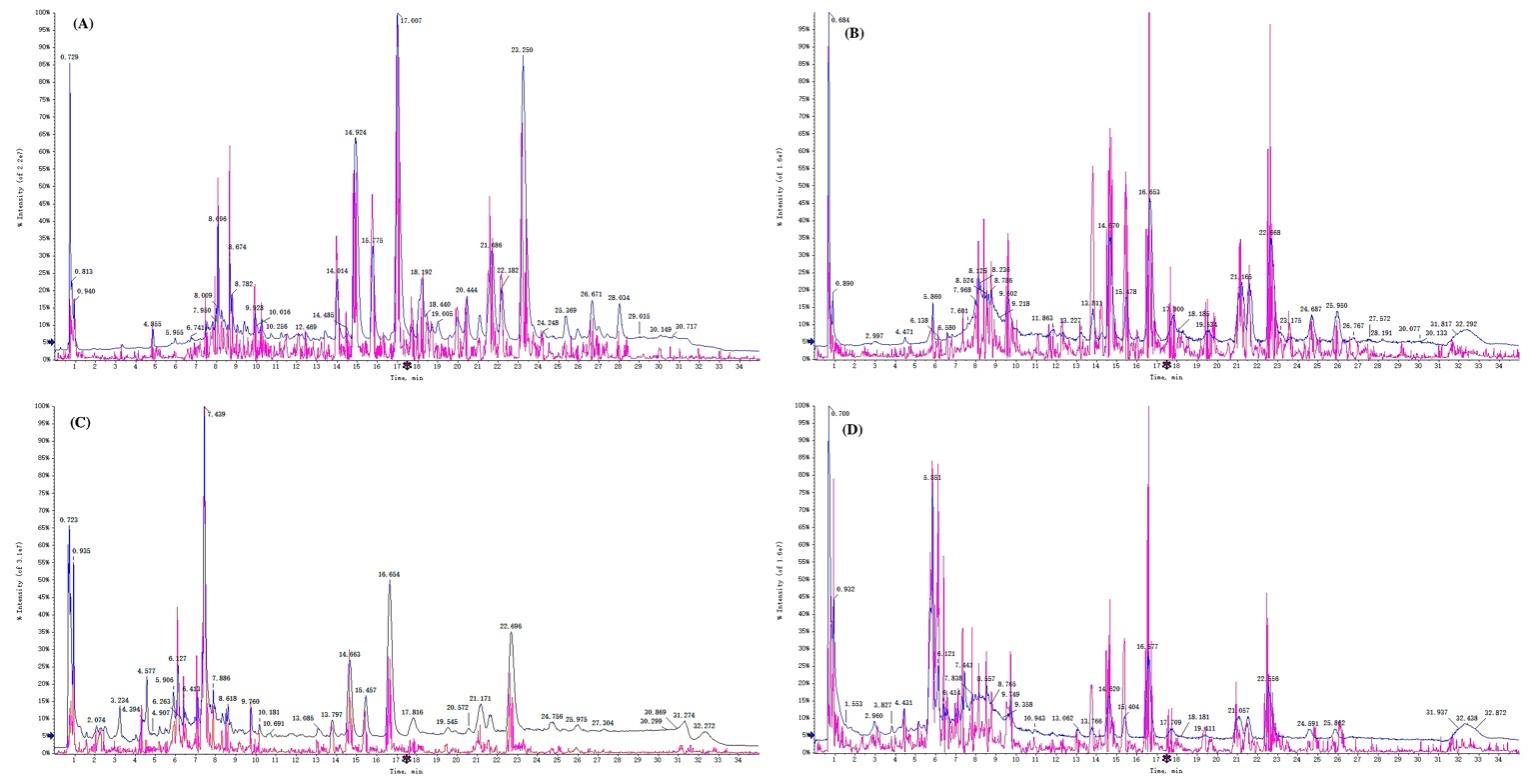


**Supplementary Figure 2.** Representative TIC chromatograms of the plasma samples. From control group: (A) ESI^+^, (B) ESI^-^; from model group: (C) ESI^+^, (D) ESI^-^.


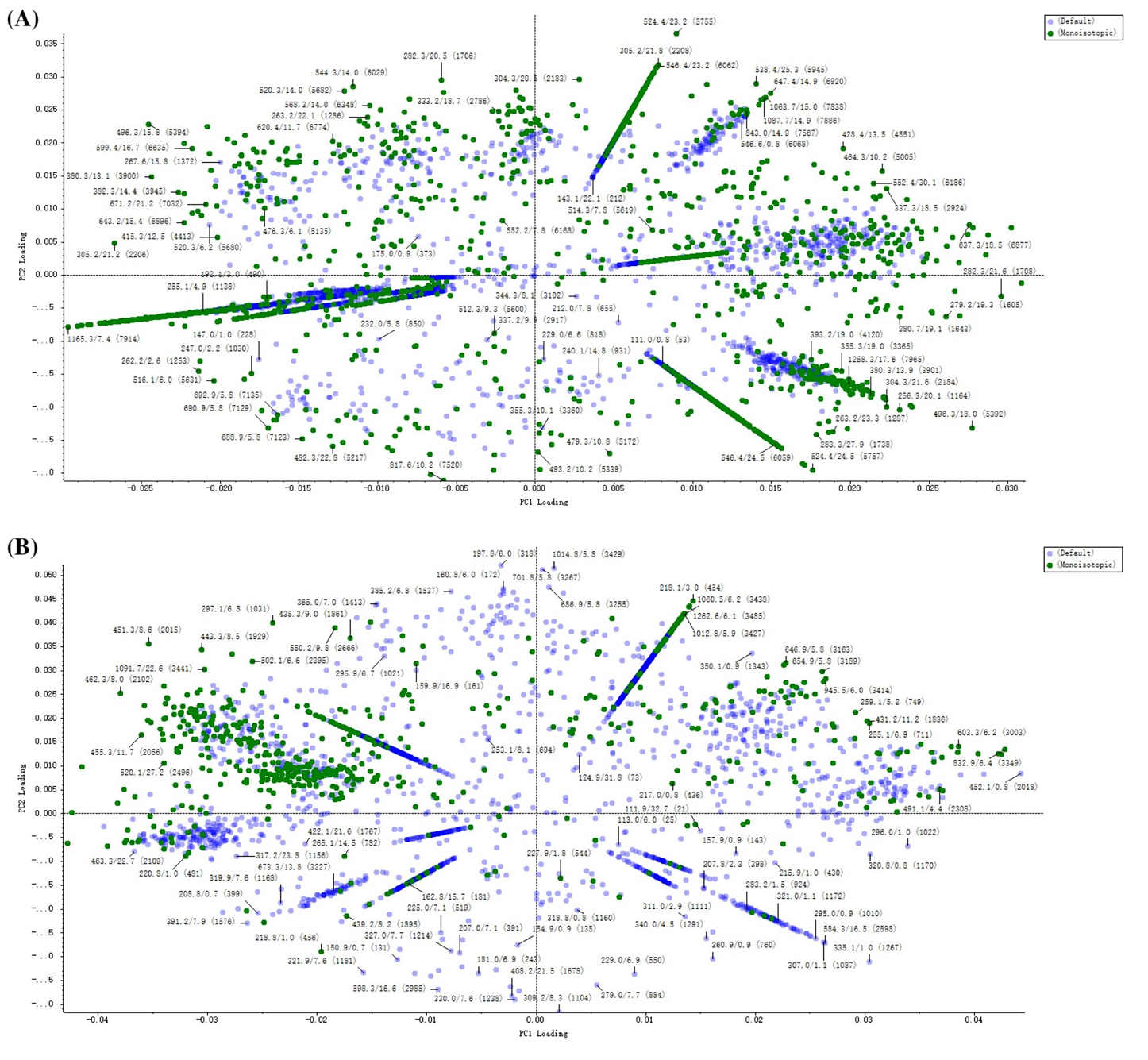


**Supplementary Figure 3.** The loading plots of PCA from control and model groups in (A) positive and (B) negative modes.


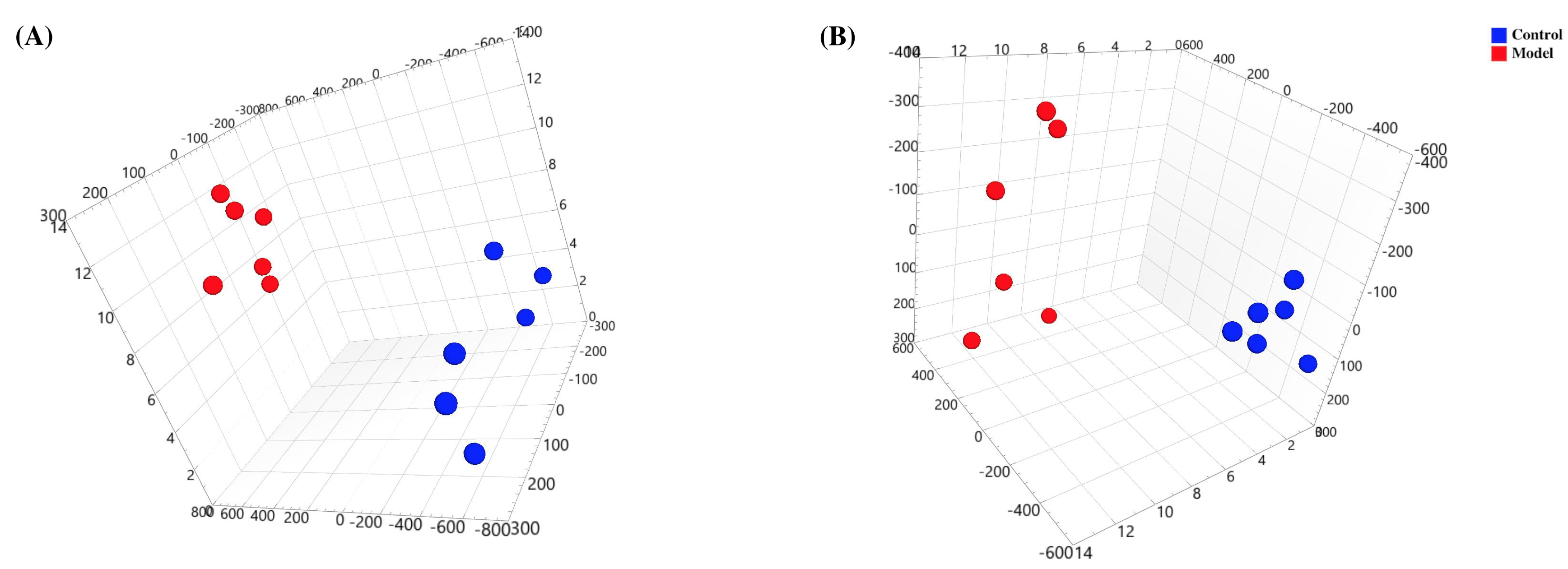


**Supplementary Figure 4.** The 3D OPLS-DA score plots of the plasma samples from control and model groups: (A) ESI^+^, *R^2^X*=0.924, *R^2^Y*=0.999, *Q^2^*=0.993; (B) *R^2^X*=0.826, *R^2^Y*=0.995, *Q^2^*=0.986.


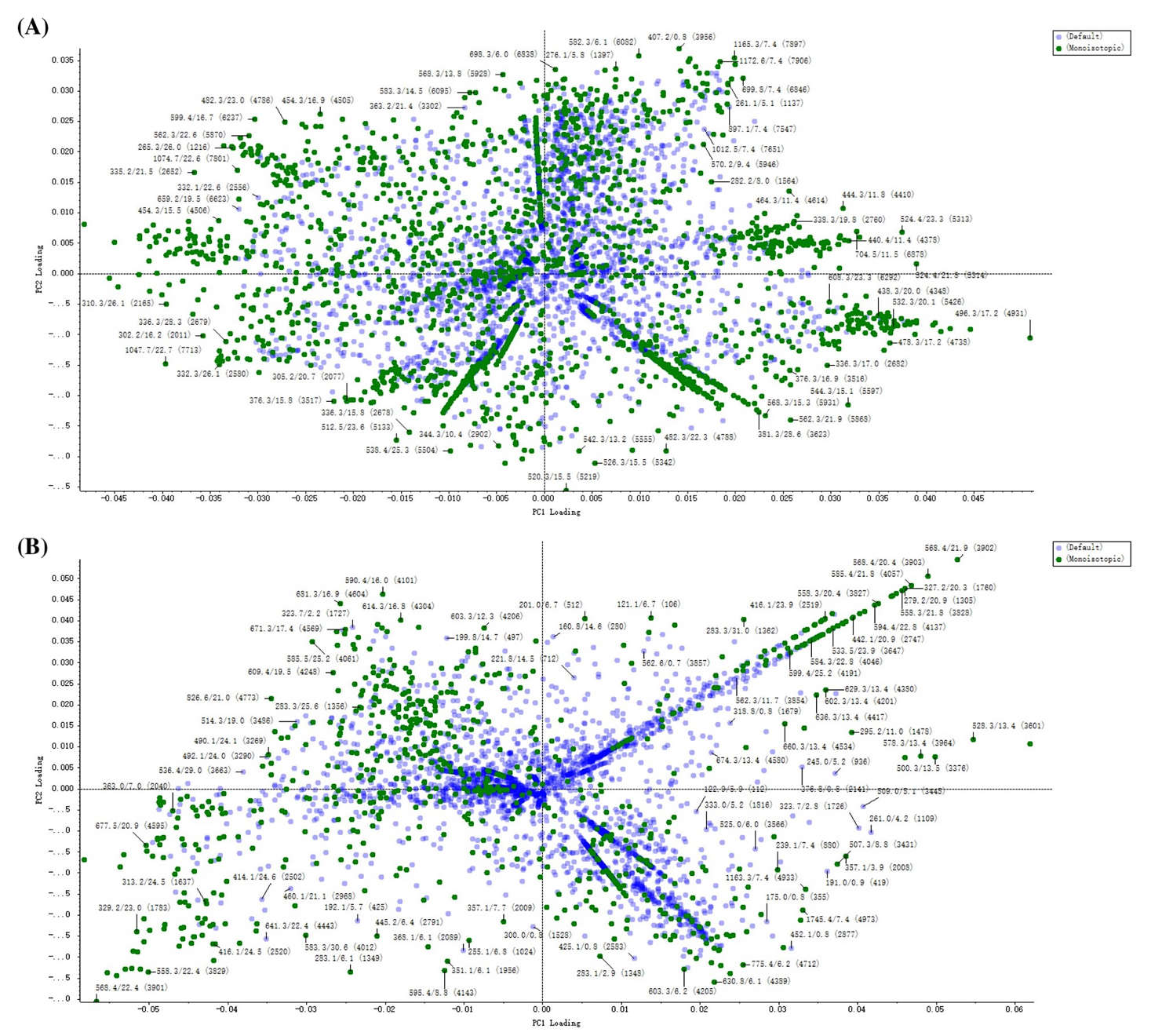


**Supplementary Figure 5.** The loading plots of PCA from model and treatment groups in (A) positive and (B) negative modes.

**
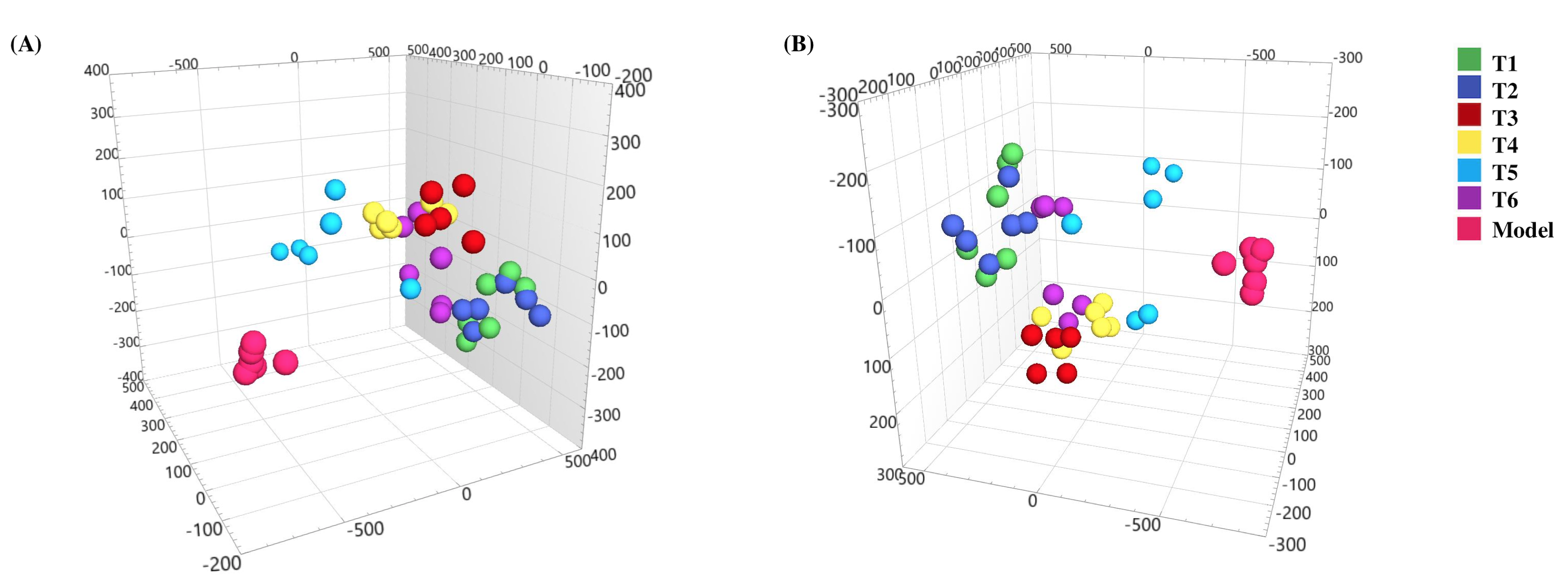
**

**Supplementary Figure 6.** The 3D OPLS-DA score plots of the plasma samples from model and treatment groups: (A) ESI^+^, *R^2^X*=0.861, *R^2^Y*=0.592, *Q*^2^=0.597; (B) *R^2^X*=0.901, *R^2^Y*=0.641, *Q*^2^=0.530.


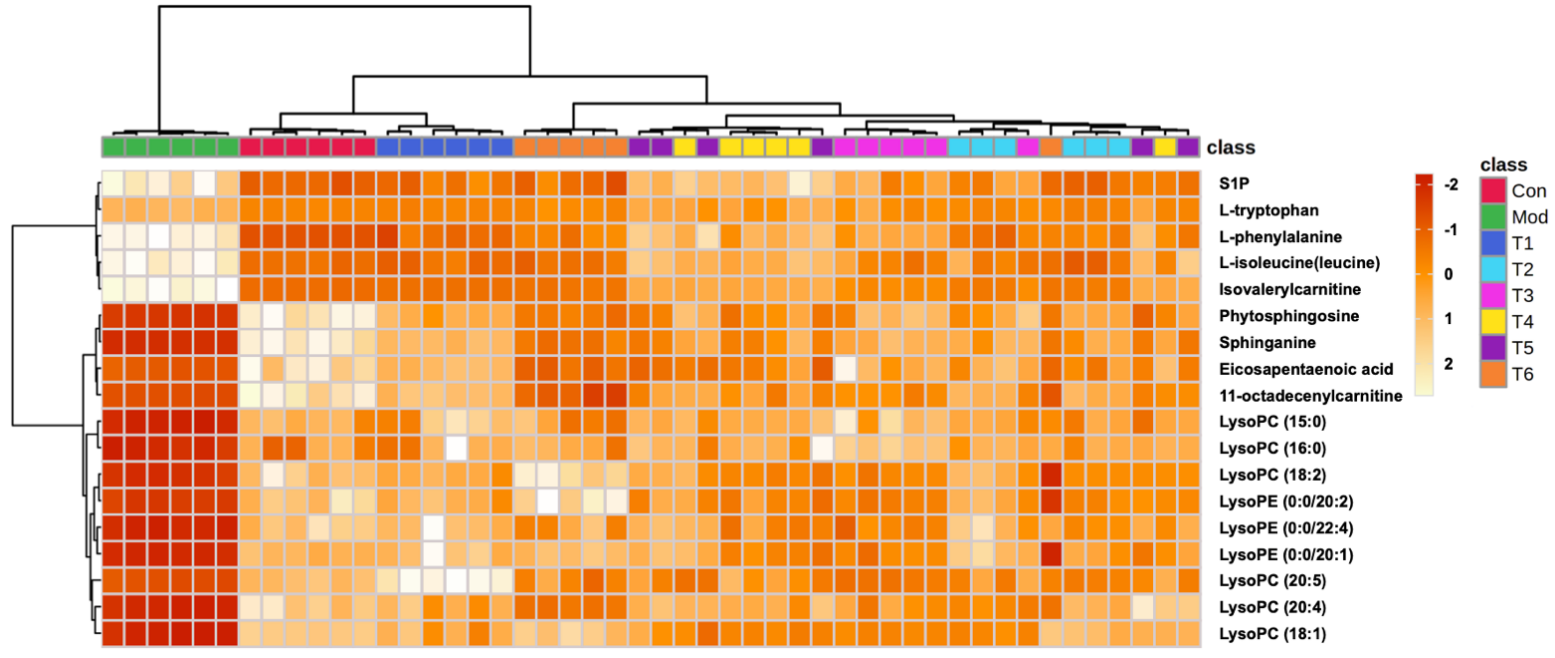


**Supplementary Figure 7.** Heatmap with hierarchical clustering analysis showed the change of biomarkers between each sample.


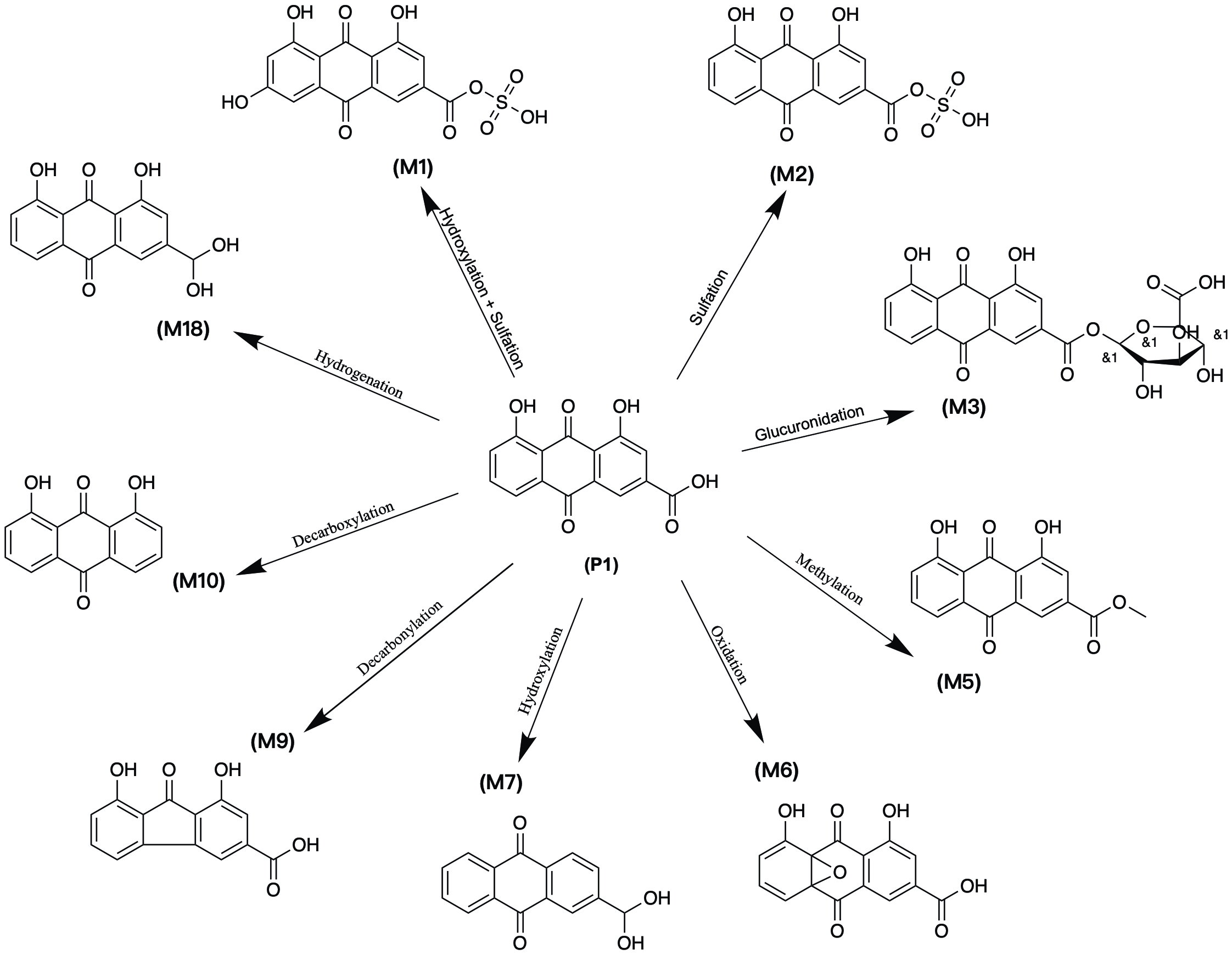


**Supplementary Figure 8.** The proposed metabolic pathways of rhein in NHBS rat plasma.
